# Ester-Modified Cyclometalated Iridium(III) Complexes as Mitochondria-Targeting Anticancer Agents

**DOI:** 10.1038/srep38954

**Published:** 2016-12-13

**Authors:** Fang-Xin Wang, Mu-He Chen, Xiao-Ying Hu, Rui-Rong Ye, Cai-Ping Tan, Liang-Nian Ji, Zong-Wan Mao

**Affiliations:** 1 MOE Key Laboratory of Bioinorganic and Synthetic Chemistry, School of Chemistry, Sun Yat-Sen University, Guangzhou 510275, China

## Abstract

Organometallic iridium complexes are potent anticancer candidates which act through different mechanisms from cisplatin-based chemotherapy regimens. Here, ten phosphorescent cyclometalated iridium(III) complexes containing 2,2′-bipyridine-4,4′-dicarboxylic acid and its diester derivatives as ligands are designed and synthesized. The modification by ester group, which can be hydrolysed by esterase, facilitates the adjustment of drug-like properties. The quantum yields and emission lifetimes are influenced by variation of the ester substituents on the Ir(III) complexes. The cytotoxicity of these Ir(III) complexes is correlated with the length of their ester groups. Among them, 4a and 4b are found to be highly active against a panel of cancer cells screened, including cisplatin-resistant cancer cells. Mechanism studies *in vitro* indicate that they undergo hydrolysis of ester bonds, accumulate in mitochondria, and induce a series of cell-death related events mediated by mitochondria. Furthermore, 4a and 4b can induce pro-death autophagy and apoptosis simultaneously. Our study indicates that ester modification is a simple and feasible strategy to enhance the anticancer potency of Ir(III) complexes.

Since cisplatin was found to possess antitumor activity, metal-based anticancer complexes have gained increasing attention over the past few decades. Many non-platinum metal complexes, such as copper, ruthenium and osmium complexes, show promising anti-proliferative activities^1^,^2^. As demonstrated by Sadler, Meggers and Ma *et al*., organometallic Ir(III) complexes can also exert potent anticancer activities through catalyzing cellular redox reactions, inhibiting protein activities or protein-protein interactions^3^,^4^,^5^,^6^,^7^.

Phosphorescent cyclometalated Ir(III) complexes are regarded as excellent probes for biological imaging and sensing, due to their outstanding photophysical properties, including relatively high quantum yields, long emission lifetimes, large Stokes shifts, two-photon absorption and high photobleaching resistance^8^,^9^,^10^. On the other hand, cyclometalated Ir(III) complexes are also considered to be potent anticancer candidates as they can target subcellular organelles, inhibit protein activities and act as photodynamic therapeutic agents^11^,^12^,^13^,^14^,^15^,^16^,^17^. Our group has endeavored to develop cyclometalated Ir(III) complexes as multifunctional theranostic agents integrating anticancer properties and imaging capabilities^14^,^15^,^16^,^17^.

As for drug optimization, it is important to improve ADMET (absorption, distribution, metabolism, excretion and toxicity)^18^. Many metal-based drugs are accompanied with poor stability and low bioavailability, which hinders their clinical application. In order to solve such problems, drugs should be modified by either hydrophilic or hydrophobic groups to enhance/modulate their biological activity. Esterification is an efficient and convenient optimization method for carboxylic acid-containing compounds, which can markedly improve their cellular uptake efficacy^19^,^20^. As carboxyl groups can undergo deprotonation, molecules bearing carboxyl groups are usually negatively charged under physiological conditions, which makes them difficult to penetrate the negatively charged cell membrane. Many marketed drugs use simple esterification to enhace oral bioavailability and absorption in the treatment of many severe diseases, such as enalapril (an angiotensin-converting enzyme inhibitor), oseltamivir (an anti-influenza drug) and MGS0210 (a glutamate receptor antagonist)^21^,^22^,^23^.

Herein, ten phosphorescent cyclometalated Ir(III) complexes containing 2,2′-bipyridine-4,4′-dicarboxylic acid (H_2_dcbpy) and its diester derivatives as ligands are designed and synthesized. Their anti-proliferative activities are evaluated on several cancer cell lines. The *in vitro* hydrolysis of the ester bonds, as well as anticancer mechanisms including subcellular localization, impact on mitochondrial integrity, elevation of reactive oxygen species (ROS), depletion of cellular ATP production, cell cycle arrest and induction of autophagy and apoptosis, are investigated in detail.

## Results and Discussion

### Synthesis and Characterization

**Figure 1:**
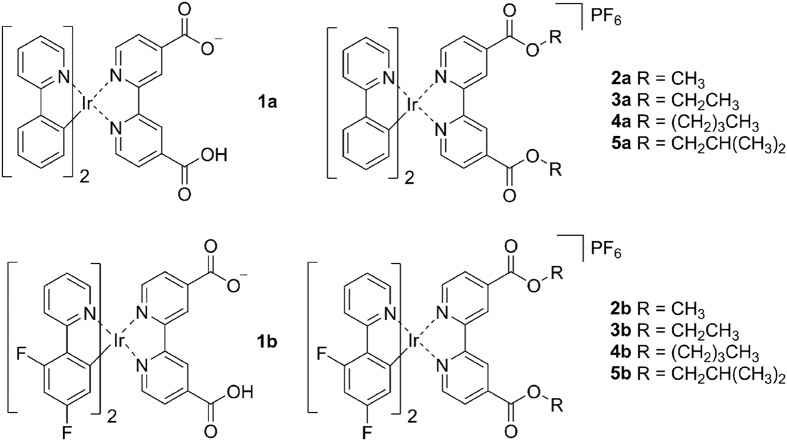
The chemical structures of cyclometalated Ir(III) complexes.

**Figure 2:**
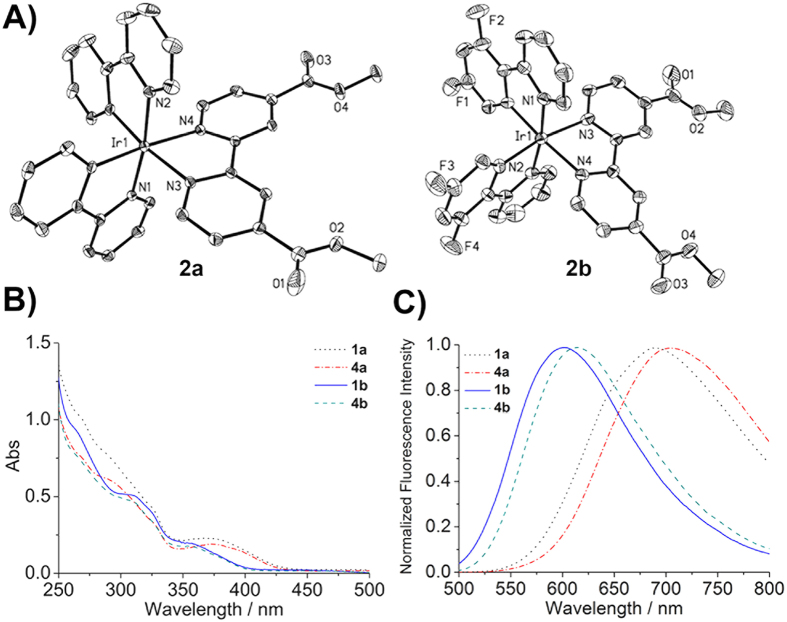
(**A**) X-ray crystal structures of **2a** and **2b**. The thermal ellipsoids are drawn at the 30% probability level. H atoms, counter ions and solvent molecules are omitted for clarity. (**B**) UV-vis absorption spectra and (**C**) emission spectra of representative Ir(III) complexes in CH_3_CN at 298 K.

The chemical structures of these Ir(III) complexes are shown in Fig. 1. Two C^N ligands, namely 2-phenylpyridine (ppy, **1a**‒**5a**) and 2-(2,4-difluorophenyl)pyridine (dfppy, **1b**‒**5b**), are utilized to tune the photophysical properties of the complexes. The ligands were prepared by reacting H_2_dcbpy with methanol, ethanol, *n*-butanol or *i*-butanol in SOCl_2_ according to reported procedures^24^. **1a** and **1b** were synthesized by refluxing the corresponding Ir(III) chloro-bridged precursors and H_2_dcbpy (0.4 mmol) in CH_2_Cl_2_/CH_3_OH with excessive Na_2_CO_3_^25^. Other complexes were synthesized by refluxing the precursors with corresponding ligands in CH_2_Cl_2_/CH_3_OH, followed by anion exchange with NH_4_PF_6_ solution (Supplementary Fig. S1). All the complexes were purified by silica flash column chromatography eluting with CH_2_Cl_2_/CH_3_OH^15^. The complexes were characterized by ESI-MS, ^1^H NMR (Supplementary Figs S2‒S11) and elemental analysis. **2a** and **2b** were also characterized by X-ray diffraction (Fig. 2A). The crystallization data are listed in Supplementary Table S1, and selected bond lengths and bond angles are listed in Supplementary Table S2.

### Photophysical Properties

The photophysical properties of Ir(III) complexes in phosphate buffered saline (PBS), CH_2_Cl_2_ and CH_3_CN were investigated (Supplementary Table S3). All complexes show intense absorption bands at 250‒420 nm (Fig. 2B), which can be assigned to mixed ligand-centred (LC) transition, ligand-to-ligand charge transfer (LLCT) and singlet and triplex metal-to-ligand charge transfer (^1^MLCT and ^3^MLCT). Upon excitation at 405 nm, **1a**−**5a** and **1b**−**5b** exhibit red to yellow phosphorescent emissions. Quantum yields of Ir(III) complexes range from 0.001 to 0.464, and phosphorescent lifetimes fall between 4.4 and 700.8 ns in different solvents at room temperature. Higher quantum yields and longer lifetimes are observed in organic solvents than in aqueous phase. The environmentally sensitive emission properties are commonly observed for cyclometalated Ir(III) complexes, which are considered to be advantageous for bioimaging applications^26^. **1b**−**5b** show blue shifts compared with **1a**−**5a**, due to the electron-withdrawing F atoms on dfppy (Fig. 2C and Supplementary Fig. S13). The energy gap between the highest occupied molecular orbital (HOMO) and the lowest unoccupied molecular orbital (LUMO) is increased by fluorine substitutions^25^.

### Hydrolysis by Esterase *in vitro*

Ester-modified compounds have been used as prodrugs widely. They can be hydrolysed by a variety of esterases selectively or non-selectively, to release active pharmacophores^27^. Herein, porcine liver esterase (PLE) was used as a model to investigate the hydrolytic process of the ester bonds in Ir(III) complexes. The hydrolysis of ten Ir(III) complexes by PLE were detected by ESI-MS (Supplementary Fig. S14)^28^. Equal amounts of Ir(III) complexes (20 μM, 100 μL) were incubated with PLE (0.6 units) in Tris-HCl solution (10 mM, pH 7.4) for 2 h. The mass spectra peaks of **1a** (*m/z* 745) or **1b** (*m/z* 817) remain unchanged. Other complexes with ester substituents show peaks assigned to intact complexes as well as peaks corresponding to hydrolytic products. The results indicate that complexes **2a**‒**5a** and **2b**‒**5b** can undergo hydrolysis in the presence of esterase.

### Determination of Log *P*_o/w_

**Figure 3:**
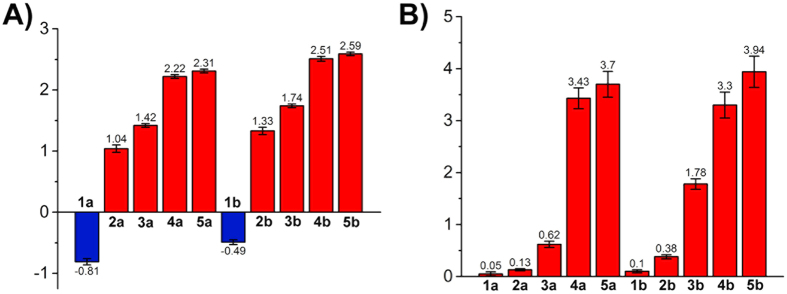
(**A**) Lipophilicity (log *P*_o/w_) of the Ir(III) complexes. (**B**) Cellular uptake of the Ir(III) complexes determined by ICP-MS. Cells were incubated with Ir(III) complexes (20 μM) at 310 K for 1 h.

According to the Lipinski’s rule, appropriate hydrophilicity and lipophilicity are of vital importance for drugs^29^. Excessive hydrophilicity decreases penetration ability of a drug to cross phospholipid layer membrane. On the contrary, high lipophilicity means poor water solubility, which hinders biomedical applications^30^. The lipophilicity, as represented by the log *P*_o/w_ values, was determined by the shake-flask method according to a literature procedure^31^. The log *P*_o/w_ values of these Ir(III) complexes fall between −0.81 and 2.59 (Fig. 3A). Complexes with longer ester chains or F atoms possess higher log *P*_o/w_ values.

### Cellular Uptake Efficacy of Ir(III) complexes

It has been reported that cellular uptake efficacy of a compound is affected by many factors, such as lipophilicity, polarization and surface area^32^. As iridium is an exogenous element, its cellular uptake level can be quantitatively recorded by ICP-MS (inductively coupled plasma-mass spectrometry)^15^. A549 (human lung adenocarcinoma epithelial) cells were incubated with Ir(III) complexes (20 μM) for 1 h and analysed. Cell penetration capability of these complexes is correlated with the length of ester group and lipophilicity (Fig. 3B). **1a** and **1b** show lowest penetration ability to across the cell membrane with lipid bilayers, which can be attributed to their low lipophilicity. High cellular uptake levels are observed for **4a**, **4b**, **5a** and **5b** with longer ester chains.

### Cytotoxicity of Ir(III) Complexes *in vitro*

The cytotoxicity was evaluated in A549, A549R (cisplatin-resistant A549), HeLa (human cervical cancer), HepG2 (human hepatocellular liver carcinoma) and MCF-7 (human breast adenocarcinoma) cells. Cisplatin was also tested as a positive control. Generally, the anti-proliferative efficacy of Ir(III) complexes is in the following order: **4a **≈ **4b **> **5a **≈ **5b **> **3b **> **3a **> **2b **> **2a **> **1a **≈ **1b**, which corresponds to their lipophilicity and cellular uptake efficacy (Table 1). **1a** and **1b** are inactive against all the cell lines tested. Complexes with longer ester substitutions (**4a**, **5a**, **4b** and **5b**) show much higher cytotoxicity than the other complexes. Especially, **4a** (IC_50_ = 1.7 μM) and **4b** (IC_50_ = 2.3 μM) are approximately 13.9−fold and 10.3−fold more potent than cisplatin (IC_50_ = 23.7 μM) against A549 cells, respectively. **4a** and **4b** are also active in A549R cells, which may indicate that they can overcome cisplatin resistance. The results indicate that ester modification can modulate lipophilicity and anti-proliferative activity of these Ir(III) complexes. For complexes **1a** and **1b**, converting the carboxyl acid groups into ester groups can facilitate cellular uptake and improve their anti-proliferative effects. As carboxyl groups are usually deprotonated at physiological pH, the phenomena may be partially attributed to the alteration of molecular charge from neutral/negative to positive under physiological conditions.

### Membrane Transport Pathway and Intracellular Fate

**Figure 4:**
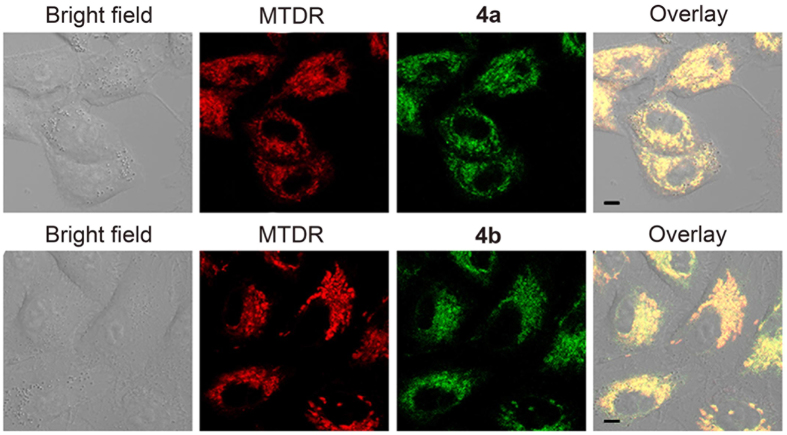
(**A**) Colocalization of **4a** and **4b** with MTDR. Cells were incubated with **4a** (5 μM) or **4b** (5 μM) for 15 min, and then stained with MTDR (100 nM) for 15 min. The excitation wavelength of complexes was 405 nm, and emission was collected at 630‒690 nm (**4a**) and 540‒600 nm (**4b**). The excitation wavelength of MTDR was 543 nm, and emission was collected at 645‒685 nm. Scale bars: 5 μm.

**4a** and **4b** were chosen for further mechanism studies because of their relatively high anticancer potency. By virtue of their intrinsic phosphorescence, the cellular uptake behaviours of Ir(III) complexes can be investigated semi-quantitatively by confocal microscopy. Moreover, subcellular localization analysis can provide further hints for the investigation of anticancer mechanisms^17^. Confocal microscopic images show that both **4a** (5 μM) and **4b** (5 μM) can penetrate into A549 cells rapidly in 30 min and mainly retain within cytoplasm (Supplementary Fig. S15). Mito-tracker^®^ Deep Red FM (MTDR), a commercial mitochondrial fluorescent probe, was co-incubated with Ir(III) complexes to further determine subcellular localization (Fig. 4)^15^. High level of colocalization can be observed for **4a** or **4b** with MTDR.

The cellular uptake mechanism was investigated by incubating cells at low temperature or pro-treating cells with carbonyl cyanide 3-chlorophenylhydrazone (CCCP, a potent mitochondrial oxidative phosphorylation uncoupler) or chloroquine (CQ, an endocytosis inhibitor)^15^. A549 cells pre-treated with CCCP (10 μM) or incubated at 277 K show markedly reduced intracellular phosphorescence of Ir(III) complexes, while pre-treatment of the cells with CQ (50 μM) shows no obvious alteration (Supplementary Fig. S16). These results collectively indicate that **4a** and **4b** enter cells by non-endocytic energy dependent active transport. Our findings are consistent with the mechanisms reported for Ir(III) complexes with structural similarity^15^,^33^.

### Induction of Mitochondrial Dysfunction

**Figure 5:**
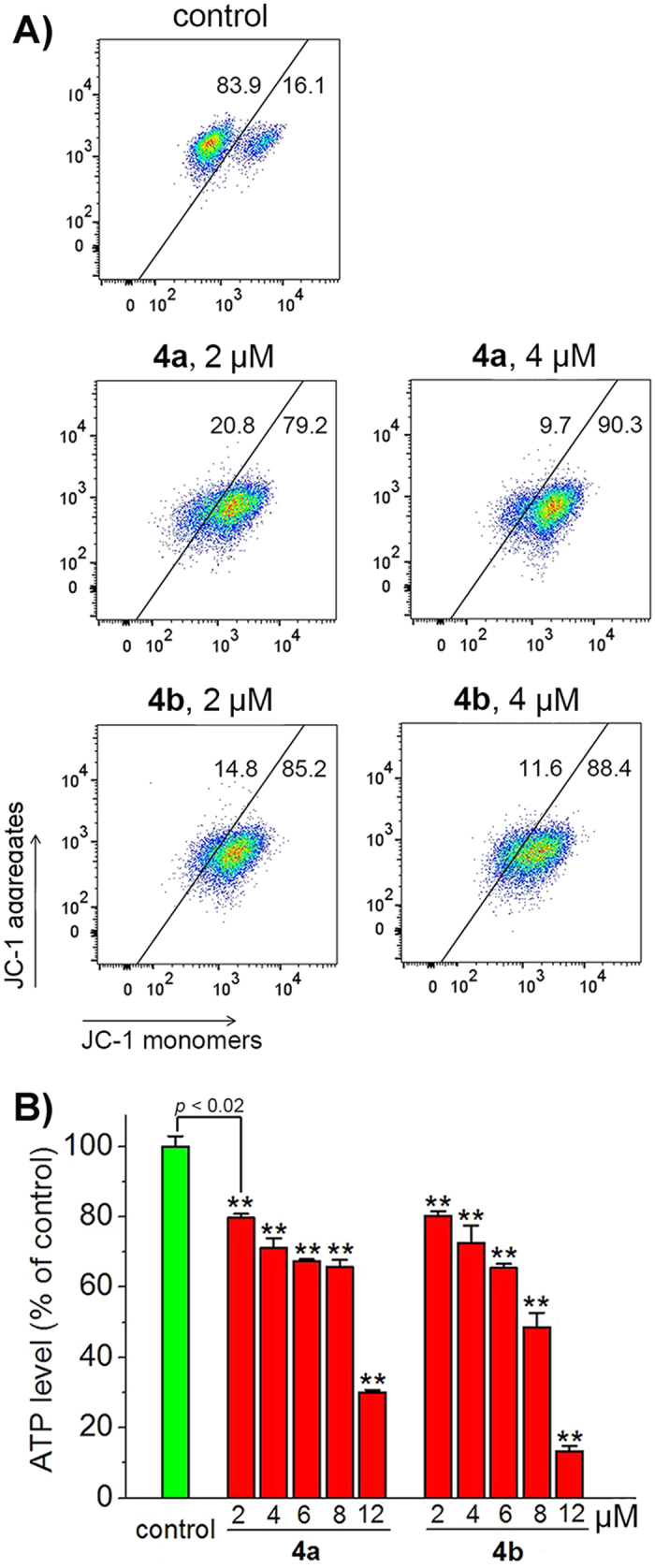
(**A**) Loss of MMP in A549 cells treated with **4a** or **4b**. Cells were incubated with Ir(III) complexes at the indicated concentrations for 6 h and then stained with JC-1. JC-1 was excited at 488 nm and monitored simultaneously at 515‒545 nm (Green) and 575‒605 nm (Red). (**B**) Depletion of cellular ATP levels in A549 cells treated with **4a** or **4b**. Cells were incubated with **4a** or **4b** at the indicated concentrations for 6 h. The luminescence intensity was measured by a microplate reader. **p < 0.02.

Based on confocal microscopy images, we further investigated the impact of **4a** and **4b** on mitochondrial integrity. Mitochondrial membrane potential (MMP) was detected by 5,5′,6,6′-tetrachloro-1,1′,3,3′-tetraethyl-imidacarbocyanine iodide (JC-1) staining. In normal mitochondria, JC-1 aggregates and shows red fluorescence. If mitochondrial membranes are depolarized, JC-1 exists as green fluorescent monomers^34^. Compared with vehicle-treated (1% dimethyl sulfoxide (DMSO)) cells, cells with depolarized mitochondria increase dose-dependently after treatment with **4a** or **4b**. The percentage of cells with MMP loss increase from 16.1 ± 0.4% to 90.3 ± 1.0% and 88.4 ± 1.1% for **4a** and **4b** respectively, after 6 h treatment at a concentration of 4 μM (Fig. 5A).

Mitochondria are the energy factory of cells, and they are vital for ATP production^35^. Cancer cells are characterized by various alterations in energy-production pathways, which makes mitochondrial metabolism a potential target for cancer therapy^36^. We used the CellTiter-Glo^®^ luminescent cell viability assay to detect the impact of Ir(III) complexes on ATP production (Fig. 5B). A dose-dependent decrease in cellular ATP concentration is observed in cells treated with **4a** or **4b**. After 12 μM Ir(III) treatments for 6 h, the ATP level decreases to 30.0 ± 0.6% and 13.3 ± 1.5% in **4a**− and **4b**−treated cells, respectively. These results indicate that **4a** and **4b** can impair mitochondrial energy metabolism in A549 cells effectively.

### Elevation of Intracellular ROS Level

**Figure 6:**
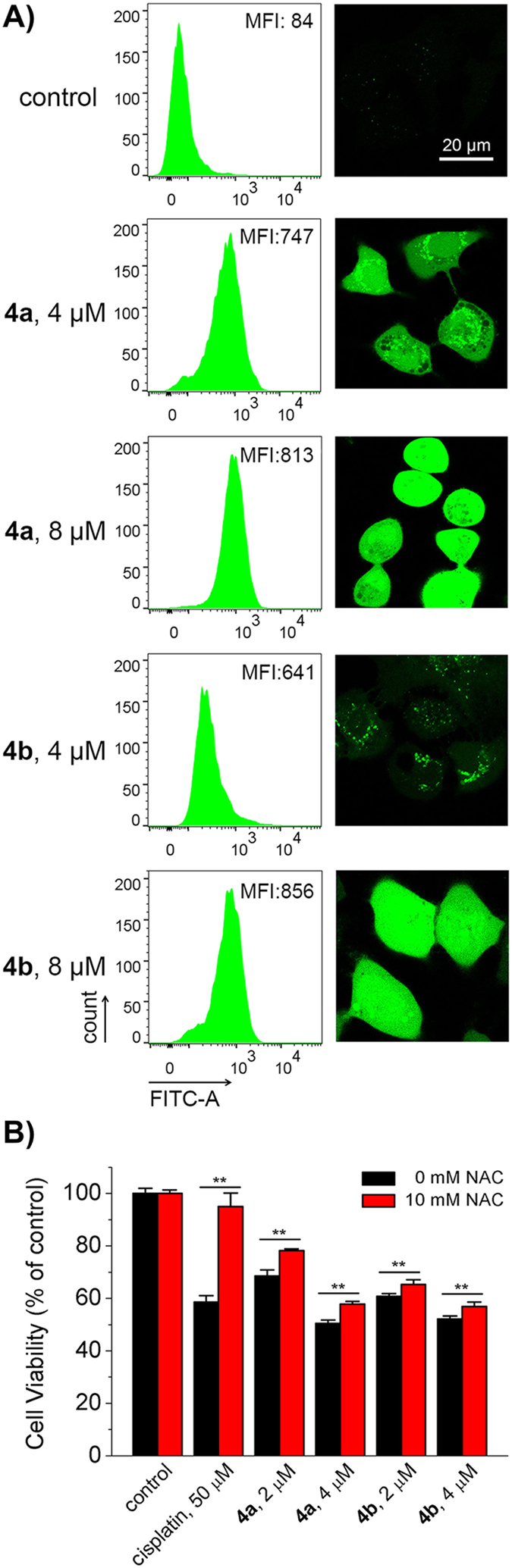
(**A**) ROS elevation in A549 cells treated with **4a** or **4b**. Cells were treated with **4a** or **4b** at the indicated concentrations for 6 h, and stained by H_2_DCFDA. Samples were detected by flow cytometry and confocal microscopy. The excitation wavelength of DCF was 488 nm. Emission was collected at 510‒540 nm. Scale bar: 20 μm. (**B**) The impact of scavenging ROS by NAC on anti-proliferative activity of **4a** or **4b**. Cells were pre-incubated with NAC for 1 h, and then treated with **4a** or **4b** for 24 h. Cell viability was measured by MTT assay. **p < 0.02.

Mitochondria are the major sites for ROS production, and conversely, they are vulnerable to ROS attack^37^. The mitochondrial damage can lead to elevation of ROS levels. Moreover, imbalanced ROS can cause cellular dysfunction^38^. As **4a** and **4b** can target to mitochondria and cause mitochondrial depolarization, their impact on intracellular ROS level was examined by 2′,7′-dichlorodihydrofluorescein diacetate (H_2_DCFDA) staining and analysed by flow cytometry as well as confocal microscopy. The non-fluorescent H_2_DCFDA can be oxidized to highly bright 2′,7′-dichlorofluorescein (DCF) by cellular ROS^39^. A dose-dependent increase in the mean fluorescence intensity (MFI) of DCF is observed in both **4a**− and **4b**−treated cells. Compared with the vehicle-treated control, 9.7− and 10.2−fold increase in intensity of DCF is observed in A549 cells treated with **4a** (8 μM, 6 h) and **4b** (8 μM, 6 h), respectively (Fig. 6A). Similar results are also obtained by confocal microscopic analysis.

In order to investigate the role of ROS in Ir(III)-induced cell death, the antioxidant *N*-acetyl-L-cysteine (NAC) was used to scavenge intracellular ROS^40^. Pre-treatment of NAC (10 mM) can diminish the anti-proliferative effects of **4a**, **4b** and cisplatin in A459 cells (Fig. 6B). Compared with the groups without NAC treatment, cell viability increases from 50.5 ± 1.3% to 57.8 ± 0.7% and 52.1 ± 1.2% to 56.9 ± 1.7% for **4a** (4 μM, 24 h) and **4b** (4 μM, 24 h), respectively. These results indicate that as a consequence of mitochondrial damage, **4a** and **4b** induce ROS-mediated cell death.

### Induction of Cell Cycle Arrest

**Figure 7:**
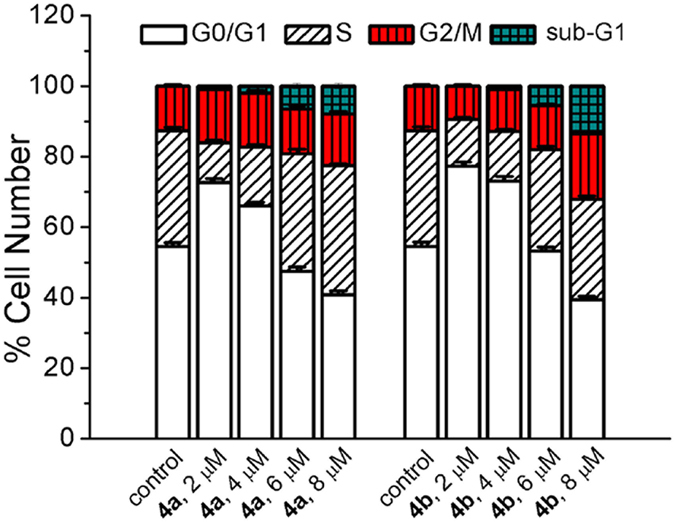
Cell cycle analysis by PI staining after A549 cells were treated with **4a** and **4b** at the indicated concentrations for 24 h.

Inhibition of membrane mitochondrial polarization, ATP depletion and ROS generation are associated with disturbance of cell cycle progression^41^,^42^. Cell cycle is tightly correlated with proliferation and development. We further studied the impact of Ir(III) complexes on cell cycle distribution. A549 cells were treated with **4a** or **4b** at different concentrations for 24 h. Cells were stained with propidium iodide (PI) and analysed by flow cytometry. The result reveals that **4a** and **4b** can elicit cell cycle arrest at G0/G1 stage at lower concentrations (2 and 4 μM), which is accompanied by a decrease in the percentage of S phase cells (Fig. 7). However, higher doses (6 and 8 μM) of Ir(III) complexes can enhance S-phase arrest. Additionally, a marked increase in the fraction of cells in sub-G1 phase (apoptotic cells with fragmented DNA) can be observed at higher doses (6 and 8 μM). These results indicate that **4a** and **4b** elicit concentration-dependent cell cycle perturbations.

### Induction of Autophagy

**Figure 8:**
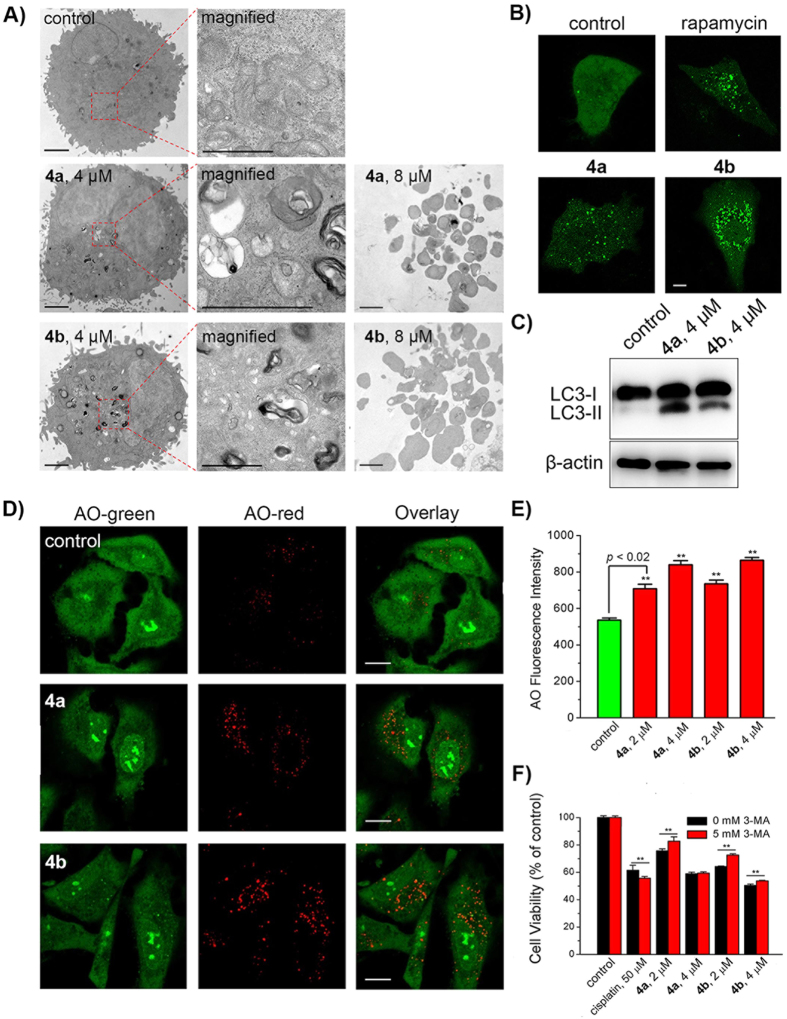
(**A**) Representative TEM images of A549 cells treated with **4a** and **4b** at the indicated concentrations for 24 h. Scale bars: 5 μm. (**B**) Representative images of A549 cells expressing GFP-LC3 treated with **4a** (4 μM), **4b** (4 μM) and rapamycin (1 μM) for 24 h. Scale bar: 5 μm. (**C**) Western blot analysis of LC3 proteins extracted from A549 cells. Cells were treated with **4a** (4 μM) and **4b** (4 μM) for 24 h. (**D**) Confocal microscopic analysis of A549 cells stained with AO after treatment with **4a** (4 μM) and **4b** (4 μM) for 24 h. AO was excited at 488 nm and monitored simultaneously at 500‒550 nm (Green) and 670‒710 nm (Red). Scale bars: 10 μm. (**E**) Mean red fluorescence intensity determined by flow cytometry in A549 cells stained with AO after treatment with **4a** or **4b** at the indicated concentrations for 24 h. AO was excited at 488 nm and emission was collected at 670‒710 nm. (**F**) The impact of 3-MA on anti-proliferative activity of cisplatin, **4a** and **4b**. Cells were pre-treated with 3-MA for 1 h and then incubated with cisplatin, **4a** or **4b** at indicated concentrations for 24 h. **p < 0.02.

Based on morphological criteria, classical cell death pathways include Type I (apoptosis), Type II (autophagic cell death), and Type III (necrosis)^43^. First, the ultra-structural changes in A549 cells treated with Ir(III) complexes were analysed by transmission electron microscopy (TEM). A549 cells treated with **4a** (4 μM) and **4b** (4 μM) for 24 h show various multilayer structures containing cytoplasmic materials at various stages of degradation, which is typical features of autophagy (Fig. 8A)^44^. However, formation of apoptotic bodies (late stage of apoptosis) can be seen in A549 cells treated with **4a** (8 μM) and **4b** (8 μM) at a higher dose. The results indicate that **4a** and **4b** can induce autophagy and apoptosis simultaneously.

Autophagy is characterized by the conversion of microtubule-associated protein 1 light chain 3 (LC3) from the cytoplasmic LC3-I to its membrane-bound form LC3-II^45^. The cellular localization of GFP-LC3 was examined after A549 cells were treated with **4a** (4 μM) and **4b** (4 μM) for 24 h. The well-known autophagy inducer rapamycin was used as a positive control for autophagy induction^46^. Compared with vehicle-treated control, cells treated with **4a** (4 μM) and **4b** (4 μM) at 24 h show a punctate pattern of GFP-LC3 fluorescence (GFP-LC3 dots), which is similar to the phenomenon observed in cells treated with rapamycin (1 μM) (Fig. 8B). Western blotting analysis also confirms the conversion of LC3-I to LC3-II in A549 cells treated with **4a** and **4b** (Fig. 8C).

The formation of acidic vesicular organelles (AVOs), is another morphological characteristic of autophagy^47^,^48^. The AVOs formation in A549 cells treated with **4a** and **4b** was also detected by acridine orange (AO). AO emits green fluorescence in nuclear and cytoplasm. While in acidic compartments, it emits red fluorescence^49^. The accumulation of AVOs, as indicated by the bright red punctuate staining dots in the cytoplasm, can be observed in A549 cells treated with **4a** and **4b** at a concentration of 4 μM for 24 h (Fig. 8D). The increase in mean fluorescence intensities of the red fluorescence of AO in Ir(III)-treated A549 cells recorded by flow cytometry also indicates the elevation in AVOs (Fig. 8E). These results further confirm the autophagic response induced by **4a** and **4b**.

Autophagy is a complicated biological process, which can play either pro-survival or pro-death roles in anticancer therapy^50^. 3-methyladenine (3-MA), a specific class III PI3K inhibitor that can block autophagosome formation, was used to investigate the relationship between autophagy and anti-proliferative activities of **4a** and **4b**^51^. The anti-proliferative effects are diminished by pre-treatment of A549 cells with 3-MA (5 mM) for 1 h (Fig. 8F). Cell viabilities increase by approximately 1.09−fold and 1.13−fold for **4a** (2 μM, 24 h) and **4b** (2 μM, 24 h), respectively. In contrast, pre-treatment of cells with 3-MA can enhance cell death induced by cisplatin, which is consistent with literature reports^51^. These results collectively indicate that Ir(III) complexes can induce pro-death autophagy.

### Induction of Apoptosis

Based on previous results obtained by TEM, we further characterized apoptosis induced by **4a** and **4b**. Apoptosis is featured by a range of defined morphological events (e.g., cell shrinkage, nuclear fragmentation and chromatin condensation) and biological events (e.g., activation of caspase family proteases and externalization of phosphatidylserine)^52^,^53^,^54^.

Firstly, the morphological changes in A549 cells treated with **4a** and **4b** was examined by 2′-(4-ethoxyphenyl)-5-(4-methyl-1-piperazinyl)-2,5′-bi-1*H*-benzimidazole trihydrochloride (Hoechst 33342) staining. Vehicle-treated cells show normal morphology and homogeneous nuclear staining pattern (Supplementary Fig. S17). After treatment with **4a** or **4b**, apoptotic cells increase obviously in a concentration-dependent manner and exhibit typical apoptotic morphological changes including cell shrinkage, membrane bubbling, condensed chromatin, nuclear fragmentation and formation of apoptotic bodies.

**Figure 9:**
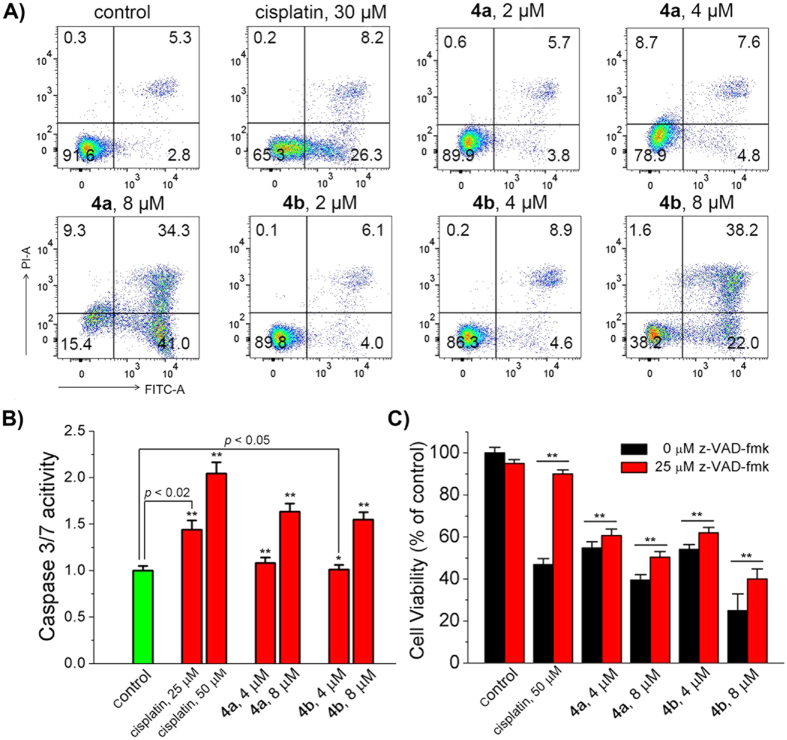
(**A**) Flow cytometric quantification of Annexin V-FITC and PI double labelled A549 cells after treatment with cisplatin, **4a** and **4b** for 24 h at the indicated concentrations. The excitation wavelength was 488 nm and the emission was monitored at 525 ± 20 nm for Annexin V-FITC and 615 ± 20 nm for PI. (**B**) Activation of caspase 3/7 in A549 cells by Ir(III) treatment. The cells were incubated with cisplatin, **4a** and **4b** at the indicated concentrations for 24 h. (**C**) The effects of the pan-caspase inhibitor z-VAD-fmk on Ir(III)-induced cell death. Cells were pre-incubated with z-VAD-fmk for 1 h and then treated with cisplatin, **4a** and **4b** for 24 h. **p < 0.02.

Annexin V-FITC/PI double staining can differentiate early apoptotic (Annexin V-FITC positive and PI negative), necrotic and late apoptotic (Annexin V-FITC positive and PI positive), and viable (Annexin V-FITC negative and PI negative) cells. Treatment of A549 cells with **4a** or **4b** leads to a dose-dependent increase in the percentage of cells in both early apoptotic and late apoptotic phases (Fig. 9A). Apoptosis induced by **4a** and **4b** is not very obvious at relatively lower concentrations (2 μM and 4 μM). At a concentration of 8 μM, the percentage of apoptotic cells increases from 8.1% to 75.3% and 60.2% for **4a** and **4b**, respectively. The apoptosis-inducing capability of **4a** and **4b** is much higher than that of cisplatin.

Caspases, a family of cysteine proteases, are central regulators of apoptosis^55^,^56^. Caspase-Glo^®^ 3/7 assay was used to investigate caspase-3/7 activation induced by **4a** and **4b** (Fig. 9B). A slight increase in the activity of caspase-3/7 is observed in **4a**− and **4b**−treated cells at a concentration of 4 μM. Compared with control cells, conspicuous increases in caspase-3/7 activity are detected in cells treated with **4a** (by 1.6−fold) and **4b** (by 1.5−fold) at a concentration of 8 μM. Moreover, pre-treatment of A549 cells with the pan-caspase inhibitor, z-Val-Ala-Asp-fmk (z-VAD-fmk), can impair the efficacy of cell death induced by Ir(III) complexes (Fig. 9C). These results indicate that **4a** and **4b** can induce caspase-dependent apoptotic cell death at a relatively higher concentration.

## Conclusion

In this work, ten phosphorescent cyclometalated Ir(III) complexes containing H_2_dcbpy and its diester derivatives have been synthesized and characterized. The impact of ester modifications on photophysical properties, lipophilicity, cellular uptake ability and anticancer activity of the iridium complexes are investigated in detail. Among them, **4a** and **4b** show the highest anti-proliferative activities towards several cancer cell lines. **4a** and **4b** mainly localize to mitochondria and initiate a series of events associated with mitochondrial dysfunction including ATP depletion, loss of MMP and elevation of ROS. Further mechanism studies indicate that **4a** and **4b** can cause concentration-dependent cell cycle arrest, pro-death autophagy and caspase-dependent apoptosis in A549 cells. Our study demonstrates that ester modification is an effective strategy for structural optimization of anticancer cyclometalated Ir(III) complexes. Synergistic effects may be achieved by conjugating Ir(III) complexes with other drugs by ester linkage, which is underway in our laboratory.

## Experimental Section

### Materials

All solvents were of analytical grade (>95% purity). All buffer components were of biological grade and used without further purification. Iridium chloride hydrate, ppy, dfppy, H_2_dcbpy and NH_4_PF_6_ were purchased from Alfa Aesar. Cisplatin, rapamycin, DMSO, PLE, PI, NAC, MTT, H_2_DCFDA, JC-1, 3-MA, CCCP, CQ, AO and Annexin V-FITC apoptosis detection kit were purchased from Sigma-Aldrich. MTDR was purchased from Invitrogen. Lipofectamine^®^ 3000 was purchased from Thermo Fisher Scientific. Hoechst 33342 and z-VAD-fmk were purchased from Beyotime. Caspase-Glo^®^ 3/7 assay kit and CellTiter-Glo^®^ luminescence cell viability assay kit were purchased from Promega.

Ligands L_1_ (dimethyl 2,2′-bipyridine-4,4′-dicarboxylate), L_2_ (diethyl 2,2′-bipyridine-4,4′-dicarboxylate), L_3_ (dibutyl 2,2′-bipyridine-4,4′-dicarboxylate) and L_4_ (diisobutyl 2,2′-bipyridine-4,4′-dicarboxylate) were synthesized according to reported procedures^24^. The cyclometalated Ir(III) chloro-bridged dimers [Ir(ppy)_2_Cl]_2_ and [Ir(dfppy)_2_Cl]_2_ were prepared according to literature methods^57^,^58^. The purity of synthesized complexes was analysed via combustion analysis and was found to be ≥ 95% pure. All the tested complexes were dissolved in DMSO just before the experiments, and the final concentration of DMSO was 1% (v/v).

### Measurements

Microanalysis (C, H and N) was conducted using a VarioEL CHNS analyser. ESI-MS spectra were recorded on a Thermo Finnigan LCQ DECA XP spectrometer. The quoted *m/z* values represent the major peaks in the isotopic distribution. ^1^H NMR spectra were recorded on a Bruker Avance 400 spectrometer (Germany). Shifts were referenced relative to the internal solvent signals. UV-vis spectra were recorded on a Varian Cary 300 spectriphotometer (USA). Emission spectra were recorded on an FLS 920 combined fluorescence lifetime and steady state spectrometer at 298 K (Japan). ICP-MS of Ir(III) complexes was recorded by X Series 2 ICP-MS (Thermo Elemental Co., Ltd., USA). UV-vis absorbance and fluorescence/luminescence emission intensity were recorded by an Infinite M200 Pro microplate reader (TECAN, Switzerland). TEM images were visualised by JEM 100 CX, and photographed by the Eversmart Jazz program (Scitex, Japan). Confocal microscopy images were obtained by a LSM 710 confocal laser scanning fluorescence microscopy (Carl Zeiss, Germany). Flow cytometry analyses were recorded by a BD FACSCalibur^TM^ flow cytometer (Becton Dickinson, USA).

### Synthetic procedure of 2a‒5a and 2b‒5b

As shown in Supplementary Figure S1, these complexes were synthesized by refluxing precursor (0.2 mmol) and the corresponding ligand (0.4 mmol) in CH_2_Cl_2_/CH_3_CN (90 mL, 2:1, v/v), followed by anion exchange with saturated NH_4_PF_6_ solution and purification by silica flash column chromatography eluting with CH_2_Cl_2_/CH_3_OH (10:1; v/v).

**Ir(ppy)**_**2**_**(Hdcbpy) (1a), [Ir(ppy)**_**2**_**(L**_**2**_**)](PF**_**6**_**) (3a) and Ir(dfppy)**_**2**_**(Hdcbpy) (1b)** were synthesized by literature methods^25^,^59^.

#### [Ir(ppy)_2_(L_1_)](PF_6_) (2a)

Red solid, yield: 73.9% (270.5 mg). ^1^H NMR (400 MHz, (CD_3_)_2_SO): δ 9.34 (d, J = 0.9 Hz, 2 H), 8.27 (d, J = 8.1 Hz, 2 H;), 8.13 (dd, J = 5.7, 1.6 Hz, 2 H), 8.08 (d, J = 5.6 Hz, 2 H), 7.95 (dd, J = 12.6, 4.6 Hz, 4 H), 7.66 (d, J = 5.3 Hz, 2 H), 7.16−7.09 (m, 2 H), 7.04 (td, J = 7.6, 1.1 Hz, 2 H), 6.92 (td, J = 7.4, 1.2 Hz, 2 H), 6.17 (d, 2 H), 3.98 (s, 6 H). ESI-MS (*m/z*): [M−PF_6_]^+^ calcd for C_36_H_28_IrN_4_O_4_, 773.2; found, 773.2. Elemental analysis: calcd (%) for C_36_H_28_IrN_4_O_4_PF_6_: C, 47.11; H, 3.07; N, 6.10, found: C, 46.96; H, 2.94; N, 6.09.

#### [Ir(ppy)_2_(L_3_)](PF_6_) (4a)

Red solid, yield: 74.4% (297.3 mg). ^1^H NMR (400 MHz, (CD_3_)_2_SO): δ 9.27 (s, 2 H), 8.28 (d, J = 8.1 Hz, 2 H), 8.11 (dd, J = 28.7, 5.6 Hz, 4 H), 7.95 (t, J = 7.2 Hz, 4 H), 7.68 (d, J = 5.6 Hz, 2 H), 7.12 (t, J = 6.5 Hz, 2 H), 7.04 (t, J = 7.4 Hz, 2 H), 6.93 (t, J = 7.3 Hz, 2 H), 6.16 (d, J = 7.4 Hz, 2 H), 4.39 (t, J = 6.4 Hz, 4 H), 1.80−1.66 (m, 4 H), 1.52–1.37 (m, 4 H), 0.93 (t, J = 7.4 Hz, 6 H). ESI-MS (*m/z*): [M−PF_6_]^+^ calcd for C_42_H_40_IrN_4_O_4_, 857.3; found, 857.3. Elemental analysis: calcd (%) for C_42_H_40_IrN_4_O_4_PF_6_: C, 50.35; H, 4.02; N, 5.59; found: C, 50.46; H, 3.90; N, 5.49.

#### [Ir(ppy)_2_(L_4_)](PF_6_) (5a)

Red solid, yield: 74.8% (298.9 mg). ^1^H NMR (400 MHz, (CD_3_)_2_SO): δ 9.24 (s, 2 H; H9), 8.28 (d, J = 8.2 Hz, 2 H), 8.18 (dd, J = 5.7, 1.5 Hz, 2 H), 8.10 (d, J = 5.6 Hz, 2 H), 7.95 (dd, J = 12.0, 4.8 Hz, 4 H), 7.69 (d, J = 5.7 Hz, 2 H), 7.19−7.09 (m, 2 H), 7.08−7.01 (m, 2 H), 6.93 (td, J = 7.5, 1.1 Hz, 2 H), 6.18 (d, J = 7.0 Hz, 2 H), 4.25−4.12 (m, 4 H), 2.17−1.96 (m, 2 H), 0.99 (t, J = 6.6 Hz, 12 H). ESI-MS (*m/z*): [M−PF_6_]^+^ calcd for C_42_H_40_IrN_4_O_4_, 857.3; found, 857.3. Elemental analysis: calcd (%) for C_42_H_40_IrN_4_O_4_PF_6_: C, 50.35; H, 4.02; N, 5.59; found: C, 50.16; H, 3.93; N, 5.62.

#### [Ir(dfppy)_2_(L_1_)](PF_6_) (2b)

Yellow solid, yield: 73.5% (290.0 mg). ^1^H NMR (400 MHz, (CD_3_)_2_SO): δ 9.40 (s, 2 H),8.30 (d, J = 8.4 Hz, 2 H), 8.17–8.10 (m, 4 H), 8.05 (dd, J = 12.3, 4.7 Hz, 2 H), 7.74 (t, J = 6.2 Hz, 2 H), 7.24–7.18 (m, 2 H, H2), 7.02 (t, J = 11.0 Hz, 2 H), 5.59 (dd, J = 8.3, 2.0 Hz, 2 H), 3.99 (s, 6 H). ESI-MS (*m/z*): [M−PF_6_]^+^ calcd for C_36_H_24_IrN_4_O_4_F_4_, 845.1; found, 845.1. Elemental analysis: calcd (%) for C_36_H_24_IrN_4_O_4_PF_10_: C, 43.69; H, 2.44; N, 5.66; found: C, 43.82; H, 2.49; N, 5.59.

#### [Ir(dfppy)_2_(L_2_)](PF_6_) (3b)

Yellow solid, yield: 77.9% (316.9 mg). ^1^H NMR (400 MHz, (CD_3_)_2_SO): δ 9.36 (d, J = 6.9 Hz, 2 H), 8.30 (d, J = 8.5 Hz, 2 H), 8.17–8.10 (m, 4 H), 8.05 (dd, J = 11.9, 4.4 Hz, 2 H), 7.75 (t, J = 6.3 Hz, 2 H), 7.25–7.18 (m, 2 H), 7.02 (ddd, J = 12.1, 9.5, 2.3 Hz, 2 H), 5.60 (dd, J = 8.3, 2.3 Hz, 2 H), 4.45 (q, J = 7.1 Hz, 4 H), 1.37 (t, J = 7.1 Hz, 6 H). ESI-MS (*m/z*): [M−PF_6_]^+^ calcd for C_38_H_28_IrN_4_O_4_F_4_, 873.2; found, 873.2. Elemental analysis: calcd (%) for C_38_H_28_IrN_4_O_4_PF_10_: C, 48.25; H, 3.41; N, 5.92; found: C, 47.95; H, 3.36; N, 5.89.

#### [Ir(dfppy)_2_(L_3_)](PF_6_) (4b)

Yellow solid, yield: 74.6% (320.0 mg). ^1^H NMR (400 MHz, (CD_3_)_2_SO): δ 9.30 (s, 2 H), 8.31 (d, J = 8.6 Hz, 2 H), 8.20–8.10 (m, 4 H), 8.05 (t, J = 8.1 Hz, 2 H), 7.72 (dd, J = 42.2, 5.6 Hz, 2 H), 7.27–7.15 (m, 2 H), 7.06–6.90 (m, 2 H), 5.61 (dd, J = 8.3, 2.2 Hz, 2 H), 4.41 (t, J = 6.4 Hz, 4 H), 1.81–1.68 (m, 4 H), 1.51–1.38 (m, 4 H), 0.95 (t, J = 7.4 Hz, 6 H). ESI-MS (*m/z*): [M−PF_6_]^+^ calcd for C_42_H_36_IrN_4_O_4_F_4_, 929.3; found, 929.3. Elemental analysis: calcd (%) for C_42_H_36_IrN_4_O_4_PF_10_: C, 46.97; H, 3.38; N, 5.22; found: C, 46.99; H, 3.37; N, 5.22.

#### [Ir(dfppy)_2_(L_4_)](PF_6_) (5b)

Yellow solid, yield: 75.8% (325.3 mg). ^1^H NMR (400 MHz, (CD_3_)_2_SO): δ 9.30 (s, 2 H), 8.30 (d, J = 8.5 Hz, 2 H), 8.19–8.12 (m, 4 H), 8.05 (dd, J = 12.0, 4.5 Hz, 2 H), 7.76 (d, J = 5.2 Hz, 2 H), 7.27–7.18 (m, 2 H), 7.03 (ddd, J = 12.1, 9.5, 2.3 Hz, 2 H), 5.60 (dd, J = 8.3, 2.3 Hz, 2 H), 4.24–4.12 (m, 4 H), 2.06 (dp, J = 13.2, 6.6 Hz, 2 H), 1.00 (d, J = 6.7 Hz, 12 H). ESI-MS (*m/z*): [M−PF_6_]^+^ calcd for C_42_H_36_IrN_4_O_4_F_4_, 929.3; found, 929.3. Elemental analysis: calcd (%) for C_42_H_36_IrN_4_O_4_PF_10_: C, 46.97; H, 3.38; N, 5.22.; found: C, 46.92; H, 3.37; N, 5.24.

### Crystallographic Structure Determination

Red crystals of 2a and yellow crystals of 2b were obtained by the diffusion of diethyl ether to the CH_2_Cl_2_ solution. X-ray diffraction measurements were performed on a Bruker Smart 1000 CCD diffractometer (Germany) with Mo Kα radiation (λ = 0.71073 Å) at 298(2) K. Crystal structures of **2a** (CCDC deposition no. 1469086) and **2b** (CCDC deposition no. 1469087) were solved by direct methods with the program SHELXL^60^. Unrefined and diffused solvent molecules were removed via application of the Squeeze function in PLATON. The structural plots were drawn using the xp package in SHELXL at a 30% thermal ellipsoids probability level.

### Hydrolysis by PLE *in vitro*

Ir(III) complexes (20 μM) were dissolved in freshly prepared Tris-HCl (10 mM, pH 7.4) buffer. Each sample contained 100 μL Ir(III) solution and 0.6 units PLE. After the samples were incubated at 298 K for 2 h, 400 μL acetone (253 K) was added into each sample to quench the enzymatic hydrolysis. Then the samples were centrifuged at 15000 × g for 10 min. The supernatant was collected and analyzed by ESI-MS.

### Determination of lipophilicity

The lipophilicity (log *P*_o/w_) was determined by the flask-shaking method and analyzed by ICP-MS. Equal amounts of PBS and *n*-octanol were shaken in the oscillator for 24 h. The mixture was separated to obtain oil and water phases. The mixtures were centrifuged at 3000 × g for 5 min. Equal volumes of *n*-octanol and PBS were added to the supernatant. The mixtures were shaken for another 24 h, and then centrifuged at 3000 × g for 5 min. The oil and water phases were separated carefully and dried under vacuum. The dried sample was dissolved by HNO_3_ (65%, 300 μL) and diluted to a final volume of 10 mL with Milli-Q water containing 10 ppb In. The Ir(III) concentration (C_o_ or C_w_) was measured by ICP-MS using In as the internal reference. The *P*_o/w_ value corresponds to C_o_/C_w_.

### Cell lines and culture conditions

HeLa, A549, A549R, MCF-7 and HepG2 cells were obtained from Experimental Animal Centre of Sun Yat-Sen University (Guangzhou, China). Cells were cultured in DMEM (Dulbecco’s modified Eagle’s medium, Gibco BRL) or RPMI 1640 (Roswell Park Memorial Institute 1640, Gibco BRL) medium, containing 10% FBS (fetal bovine serum, Gibco BRL), 100 μg/mL streptomycin and 100 U/mL penicillin (Gibco BRL). Cells were cultured in a humidified incubator, which provided an atmosphere of 95% air and 5% CO_2_ at a constant temperature of 310 K. A549R cells were cultured in medium containing cisplatin to maintain resistance.

### Cellular uptake efficacy by ICP-MS

A549 cells were cultured in 60 mm dishes and incubated for 24 h. The medium was removed and replaced with medium-DMSO (99:1, v/v) containing Ir(III) complexes (20 μM). After 1 h incubation, cells were trypsined and collected in PBS. After counted by hemocytometer, cells were digested with HNO_3_ (65%, 300 μL) at room temperature for 24 h. The solution was diluted to a final volume of 10 mL with Milli-Q water containing 10 ppb In. The iridium content was measured by ICP-MS using In as the internal reference.

### Cell viability assay

The cytotoxicity of the tested complexes towards different cell lines was determined by MTT assay. Cells were cultured in 96-well plates for 24 h. Cells were incubated with a series of concentrations of complexes for another 44 h. 20 μL MTT solution (5 mg/mL) was added into each well and incubated for another 4 h. Then culture media was removed and 150 μL DMSO was added into each well. The plate was shaken for 10 min. The absorbance at 595 nm was measured by a microplate reader.

### Co-localization assay

A549 cells were seeded in 35 mm dishes for 24 h and incubated with **4a** (5 μM) and **4b** (5 μM) for 15 min at 310 K. MTDR (100 nM) was added and the cells were incubated for another 15 min. Cells were washed with PBS for three times and visualized immediately by confocal microscopy. The excitation wavelength of Ir(III) complexes was 405 nm. The excitation wavelength of MTDR was 543 nm. Emission was collected at 600‒700 nm (**4a**), 550‒650 nm (**4b**) and 645‒685 nm (MTDR).

### Membrane transport pathway

A549 cells were seeded in 35 mm dishes for 24 h and incubated with **4a** (5 μM) or **4b** (5 μM) for 30 min at 310 K or 277 K. For the inhibition studies, cells were pre-incubated with CCCP (10 μM) or CQ (50 μM) for 1 h, and then incubated with **4a** (5 μM) or **4b** (5 μM) for 30 min at 310 K. Cell images were captured by confocal microscopy. The excitation wavelength of Ir(III) complexes was 405 nm. Emission was collected at 600‒700 nm (**4a**) and 550‒650 nm (**4b**).

### Analysis of MMP

A549 cells were seeded in 60 mm dishes and incubated with **4a** and **4b** at the indicated concentrations for 6 h. Then cells were collected and stained with JC-1 (5 μg/mL) for 15 min at 310 K. Cells were washed twice with PBS, and analysed by flow cytometry immediately. JC-1 was excited at 488 nm and monitored simultaneously at 515‒545 nm (Green) and 575‒605 nm (Red) by flow cytometry. Data were analysed using FlowJo 7.6 software (Tree Star, USA). 10, 000 events were acquired for each sample.

### ATP production assay

ATP production was measured using the CellTiter-Glo^®^ luminescence cell viability assay kit according to the manufacturer’s protocol. A549 cells were cultured in an opaque-walled 96-well plate and treated with **4a** and **4b** at the indicated concentrations for 6 h. 100 μL CellTiter-Glo^®^ reagent was added into each well containing 100 μL medium. The plate was incubated at room temperature for 30 min. Luminescence intensity was measured by a microplate reader.

### ROS detection by confocal microscopy

A549 cells were seeded in 60 mm dishes and incubated for 24 h, after which the cells were treated with **4a** and **4b** at the indicated concentrations for 6 h. Then cells were collected and incubated with 10 μM H_2_DCFDA in serum-free DMEM at 310 K for 15 min. Cells were washed twice with serum-free DMEM, and measured by confocal microscopy immediately. The excitation wavelength of DCF was 488 nm. Emission was collected at 510‒540 nm.

### ROS detection by flow cytometry

A549 cells were seeded in 60 mm dishes and incubated for 24 h. Then cells were treated with **4a** and **4b** at the indicated concentrations for 6 h. Cells were collected and stained by 10 μM H_2_DCFDA in serum-free DMEM at 310 K for 15 min. Cells were analysed by flow cytometry immediately. The excitation wavelength of DCF was 488 nm. Emission was collected at 510‒540 nm. Data of flow cytometry were analysed by FlowJo 7.6 software.

### Inhibition of ROS by NAC

A549 cells were cultured in 96-well plates and grown to confluence. Cells were pre-treated with NAC (10 μM) for 1 h, and then incubated with various concentrations of **4a** and **4b** for 24 h. Cell viability was measured by MTT assay.

### Cell cycle analysis

A549 cells were seeded in 60 mm dishes and incubated with **4a** and **4b** at the indicated concentrations for 24 h. After incubation, the cells were collected and fixed with 70% ethanol. After an overnight storage at 253 K in 70% ethanol, cells were washed twice with PBS and suspended with 500 μL of staining solution containing PI (10 μg/mL) and DNAse-free RNase (100 μg/mL), and then analysed by flow cytometry. Data of flow cytometry were analysed by ModFit LT 2.0 software (Variety Software House, Inc., USA).

### TEM analysis

A549 cells were treated with **4a** (5 μM) and **4b** (5 μM) at 310 K for 24 h. Cells were collected and fixed overnight at 277 K in phosphate buffer (pH 7.4) containing 2.5% glutaraldehyde. Cells were then treated with osmium tetroxide, and stained with uranyl acetate and lead citrate, and visualized under transmission electron microscopy (JEM 100 CX, JEOL, Tokyo, Japan). Images were photographed using the Eversmart Jazz program (Scitex).

### GFP-LC3 analysis

A549 cells were transfected with the GFP-LC3 vector using Lipofectamine^®^ 3000 (Thermo Fisher Scientific, USA). After 24 h, cells were treated with tested complexes for 24 h and observed by the confocal microscopy. Emission was collected at 500–540 nm upon excitation at 488 nm.

### Western blotting of LC3

A549 cells were seeded into 60 mm dishes and incubated for 24 h, and then exposed to complexes **4a** or **4b** for 24 h. Cells were collected and washed twice with ice-cold PBS, and lysed on ice in radioimmunoprecipitation assay (RIPA, Beyotime) containing proteases inhibitors (Roche Diagnostics GmbH, Mannheim, Germany). Equal amounts of proteins were added and separated on SDS-polyacrylamide gel electrophoresis. Proteins on the gel were transferred onto a polyvinylidenedifluoride membrane. The membrane was blocked in 20 mM Tris, 150 mM NaCl, and 0.05% Tween 20 (TBST, Sigma Aldrich, Missouri, USA) containing 5% non-fat dried milk at pH 8.0, and then incubated overnight at 277 K with the primary antibodies specific to β-actin and LC3 proteins. The membrane was incubated with corresponding horseradish peroxidase-conjugated secondary antibody after washing three times. The immunoreactivity was recorded by FluorChem M (ProteinSimple, CA, USA).

### AO staining by confocal microscopy

A549 cells were seeded in 60 mm dishes and incubated for 24 h. Then the cells were treated with **4a** or **4b** for 24 h, after which the cells were stained with AO at a final concentration of 1 μg/mL for 15 min at 310 K. Cells were washed twice by PBS, and analysed by confocal microscopy immediately. The excitation wavelength of AO was 488 nm. Emission was collected at 500–550 nm (Green) and 670–720 nm (Red).

### AO staining by flow cytometry

A549 cells were seeded in 60 mm dishes and incubated for 24 h, then cells were treated with **4a** or **4b** for 24 h. Cells were stained with AO (1 μg/mL) for 15 min at 310 K and washed twice by PBS before collected. Cells were analysed by flow cytometry immediately. The excitation wavelength of AO was 488 nm. Emission was collected at 670‒710 nm. Data were analysed by FlowJo 7.6 software.

### Inhibition of autophagy by 3-MA

Cells were cultured in a 96-well plate and grown to confluence. Cells were pre-treated with or without 3-MA (5 mM) for 1 h, then incubated with the complexes at the indicated concentrations for 24 h. Cell viability was measured by MTT assay.

### Hoechst 33342 staining

A549 cells were seeded in 35 mm dishes for 24 h and incubated with **4a** or **4b** at indicated concentrations for 24 h at 310 K, and then washed with ice-cold PBS and fixed with 4% paraformalclehyde for 30 min on ice. Cells were washed with ice-cold PBS twice. Then the cells were incubated with Hoechst 33342 (5 μL/mL in PBS) at room temperature in the dark for 10 min. The cells were washed twice with ice-cold PBS and visualized by confocal microscopy. The excitation wavelength of Hoechst 33342 was 405. Emission was collected at 440‒480 nm.

### Annexin V-FITC/PI assay

The assay was performed according to the manufacturer’s instructions. A549 cells were seeded in 60 mm dishes and incubated for 24 h. Then the cells were incubated with **4a** and **4b** at indicated concentrations at 310 K for 24 h. Cells were collected and suspended with binding buffer, then stained with Annexin V-FITC and PI for 10 min on ice in the dark. Cells were analysed by flow cytometry immediately. Data were analysed by FlowJo 7.6 software. The excitation wavelength of both Annexin V-FITC and PI was 488 nm. Emission was collected at 505–545 nm (Annexin V-TITC) and 595–635 (PI) separately.

### Caspase-3/7 activity assay

Caspase-3/7 activity was measured using Caspase-Glo^®^ Assay Kit according to the manufacturer’s protocol. A549 cells were cultured in opaque-walled 96-well plates and treated with **4a** and **4b** at the indicated concentrations for 6 h. 100 μL of Caspase-Glo^®^ reagent was added into each well containing 100 μL culture media. The mixture was incubated at room temperature for 30 min. The luminescence was measured by a microplate reader.

### Inhibition of caspases by z-VAD-fmk

Cells were cultured in 96-well plates and grown to confluence. Cells were pre-treated with z-VAD-fmk (25 μM) for 1 h, and then incubated with **4a** and **4b** at the indicated concentrations for 24 h. Cell viability was measured by MTT assay.

### Statistical analysis

All biological experiments were performed at least twice with triplicates in each experiment. Representative results are presented as the means ± standard deviations.

## Additional Information

**How to cite this article**: Wang, F.-X. *et al*. Ester-Modified Cyclometalated Iridium(III) Complexes as Mitochondria-Targeting Anticancer Agents. *Sci. Rep.* **6**, 38954; doi: 10.1038/srep38954 (2016).

**Publisher’s note:** Springer Nature remains neutral with regard to jurisdictional claims in published maps and institutional affiliations.

## Supplementary Material

Supplementary Information

## Figures and Tables

**Table 1:** IC_50_ values of Ir(III) complexes towards different cell lines *in vitro.*

Complex	IC_50_ (μM)^a^				
	HeLa	A549	A549R	HepG2	MCF-7
**1a**	>100	>100	>100	>100	>100
**2a**	35.1 ± 2.7	>100	77.6 ± 3.0	>100	68.4 ± 2.5
**3a**	15.8 ± 1.3	13.9 ± 1.2	9.1 ± 1.0	19.5 ± 1.7	23.5 ± 2.2
**4a**	2.1 ± 0.2	1.7 ± 0.1	2.1 ± 0.2	5.5 ± 0.4	5.3 ± 0.6
**5a**	2.2 ± 0.2	1.9 ± 0.2	2.2 ± 0.3	4.8 ± 0.4	3.4 ± 0.4
**1b**	>100	>100	>100	>100	>100
**2b**	24.6 ± 1.2	10.5 ± 1.0	10.2 ± 0.9	20.9 ± 1.9	35.2 ± 2.3
**3b**	7.3 ± 0.6	16.8 ± 1.2	14.1 ± 1.2	12.0 ± 1.1	19.5 ± 1.0
**4b**	1.9 ± 0.1	2.3 ± 0.2	1.7 ± 0.1	2.5 ± 0.2	4.3 ± 0.4
**5b**	2.4 ± 0.2	5.8 ± 0.4	2.7 ± 0.2	4.3 ± 0.3	8.4 ± 0.7
cisplatin	25.1 ± 2.2	23.7 ± 1.0	79.4 ± 6.1	15.8 ± 1.2	16.8 ± 1.5

^a^IC_50_ values are drug concentrations necessary for 50% inhibition of cell viability. Data are presented as means ± standard deviations obtained in at least three independent experiments.
