# Supplementary material for: Ester-Modified Cyclometalated Iridium(III) Complexes as Mitochondria-Targeting Anticancer Agents
Source: Sci Rep. 2016 Dec 13;6:38954. doi: 10.1038/srep38954 (PMC5154195; doi:10.1038/srep38954)
Supplement: Supplementary Information [file srep38954-s1.pdf]

Supplementary Information

Ester-Modified Cyclometalated Iridium(III) Complexes as Mitochondria-Targeting Anticancer Agents

Fang-Xin Wang‡, Mu-He Chen‡, Xiao-Ying Hu, Rui-Rong Ye, Cai-Ping Tan*,

Liang-Nian Ji, Zong-Wan Mao*

MOE Key Laboratory of Bioinorganic and Synthetic Chemistry, School of Chemistry,

Sun Yat-Sen University, Guangzhou, 510275, China.

*To whom correspondence should be addressed.

E-mail: cesmzw@mail.sysu.edu.cn (Z. W. Mao);

E-mail: tancaip@mail.sysu.edu.cn (C. P. Tan).

Fax: +86-2084112245;

Tel: +86-2084113788.

Table of Contents

Figure S1. Synthetic methods of Ir(III) complexes..S1

Figure S2-S11. ^{1}H NMR spectra of Ir(III) complexes...S2-S6

Figure S12. UV-vis spectra of Ir(III) complexes...S7

Figure S13. Fluorescence emission spectra of Ir(III) complexes...................................S8

Figure S14. Hydrolysis of Ir(III) complexes by PLE *in vitro*..S9

Figure S15. Confocal images of **4a** and **4b** accumulated in cytoplasma.......................S10

Figure S16. Study of cellular uptake pathways..S11

Figure S17. Hoechst 33342 staining and apoptosis induced by **4a** and **4b**...................S12

Table S1. Crystallographic data of **2a** and **2b**...S13

Table S2. Selected bond lengths (Å) and bond angles (deg) of **2a** and **2b**....................S14

Table S3. Photophysical properties of Ir(III) complexes...S15

References..S16

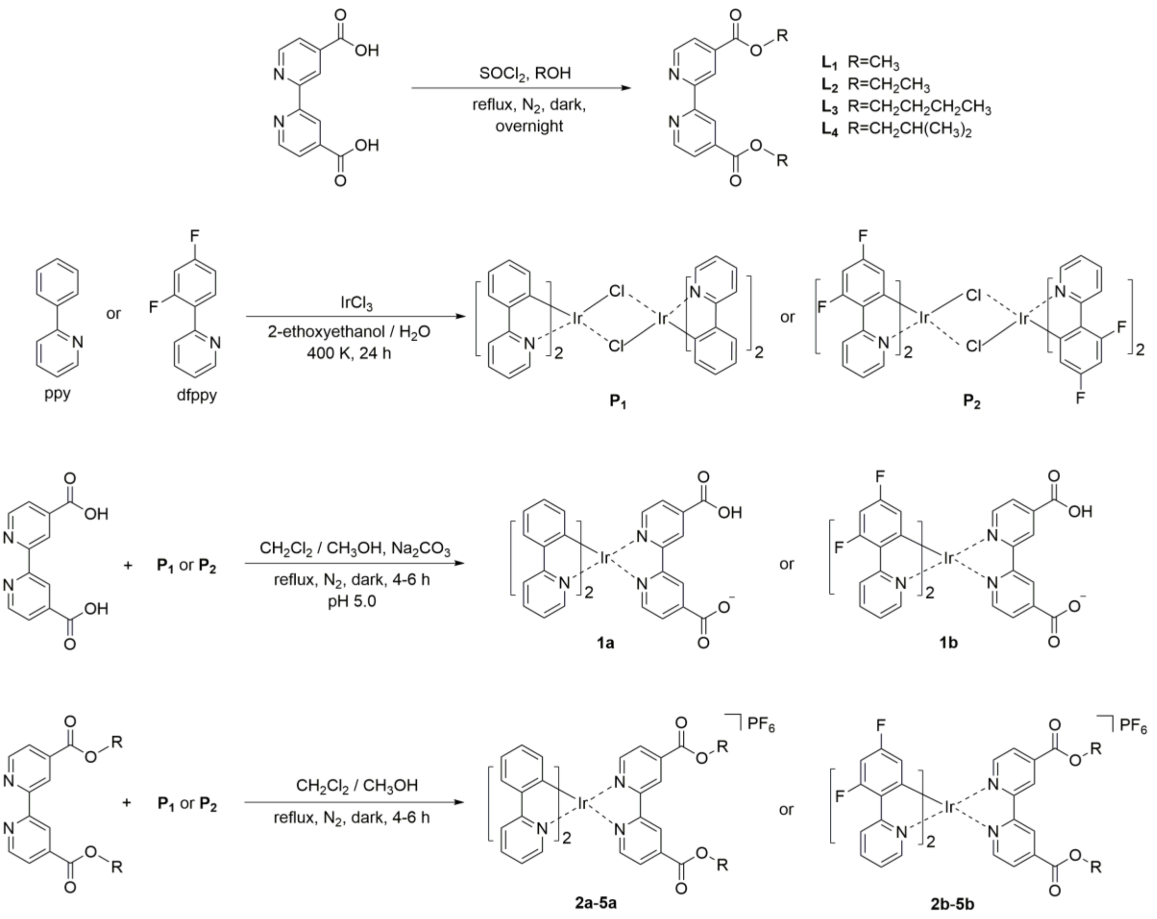

Figure S1. Synthetic methods of Ir(III) complexes.

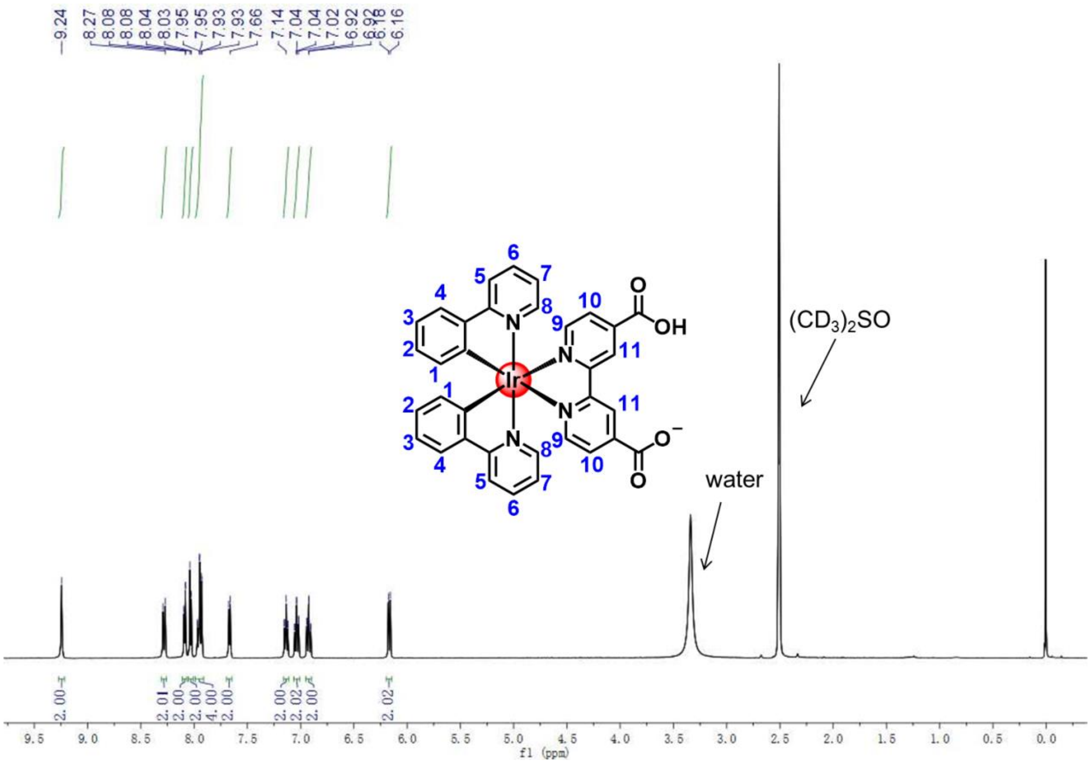

Figure S2. ¹H NMR spectrum of **1a**.

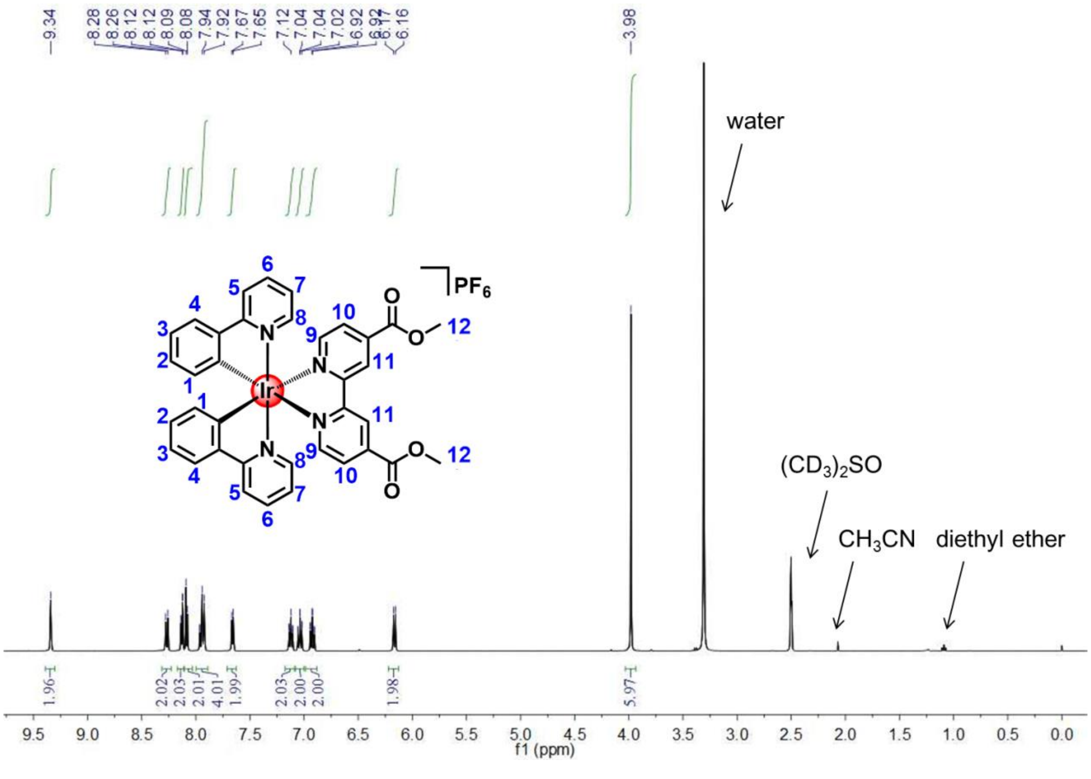

Figure S3. ¹H NMR spectrum of **2a**.

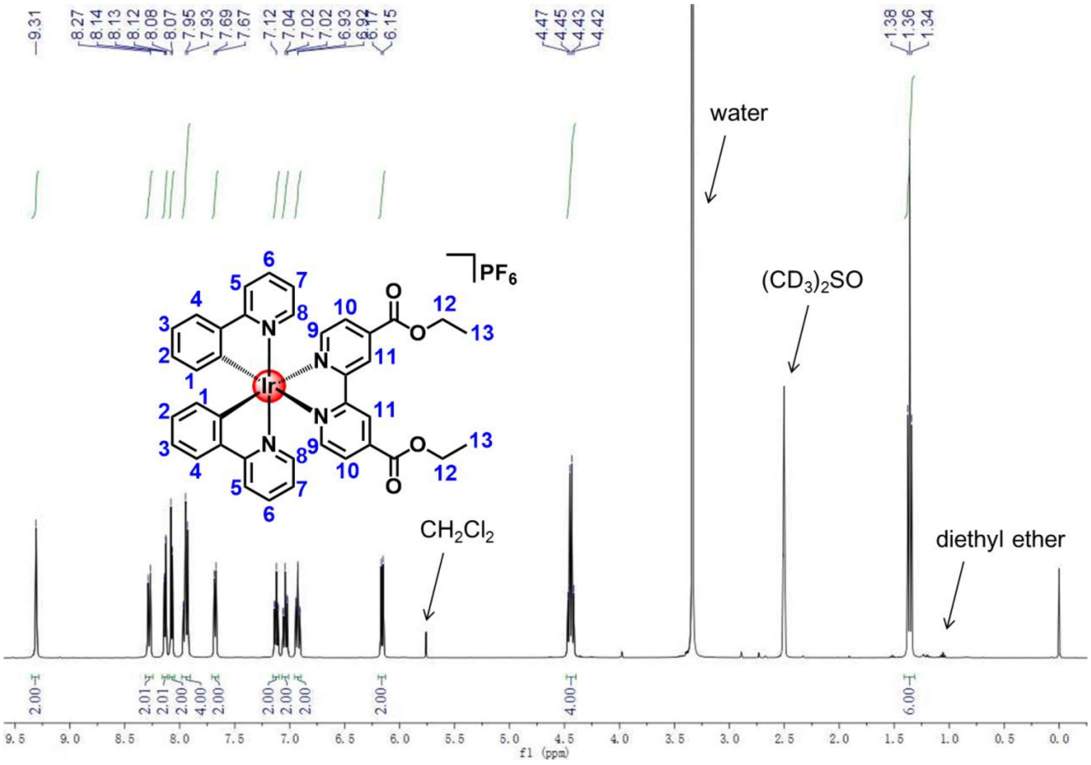

Figure S4. ^{1}H NMR spectrum of **3a**.

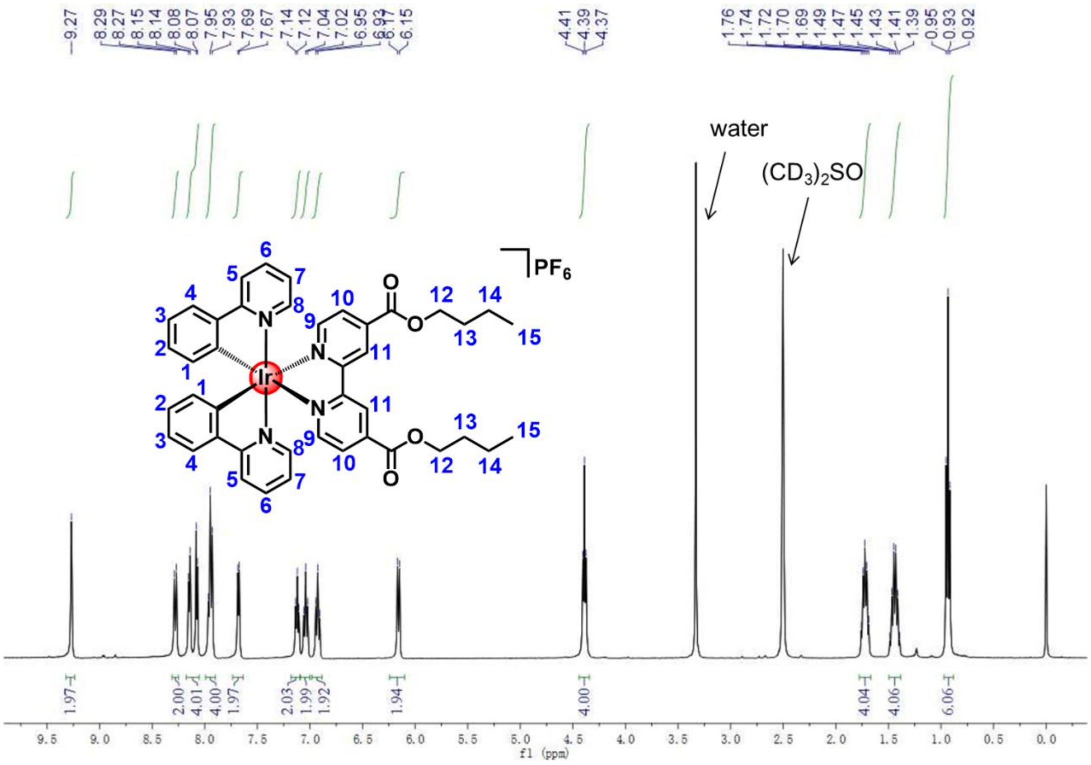

Figure S5 ^{1}H NMR spectrum of **4a**.

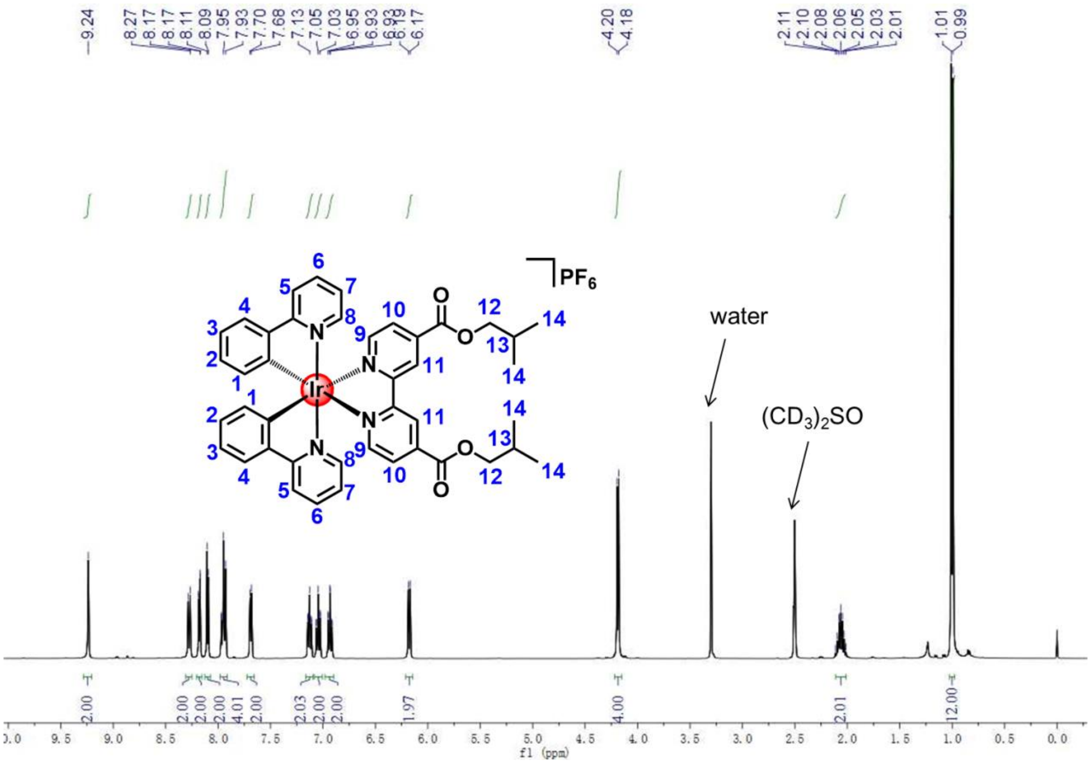

Figure S6. ^{1}H NMR spectrum of **5a**.

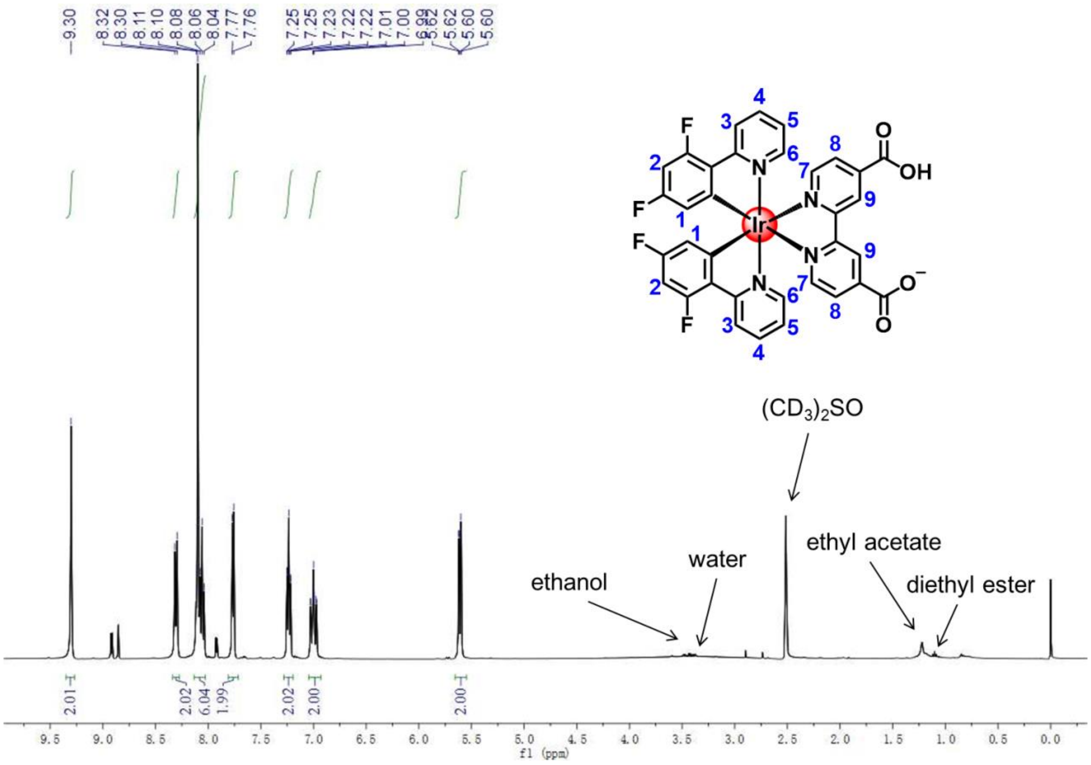

Figure S7. ^{1}H NMR spectrum of **1b**.

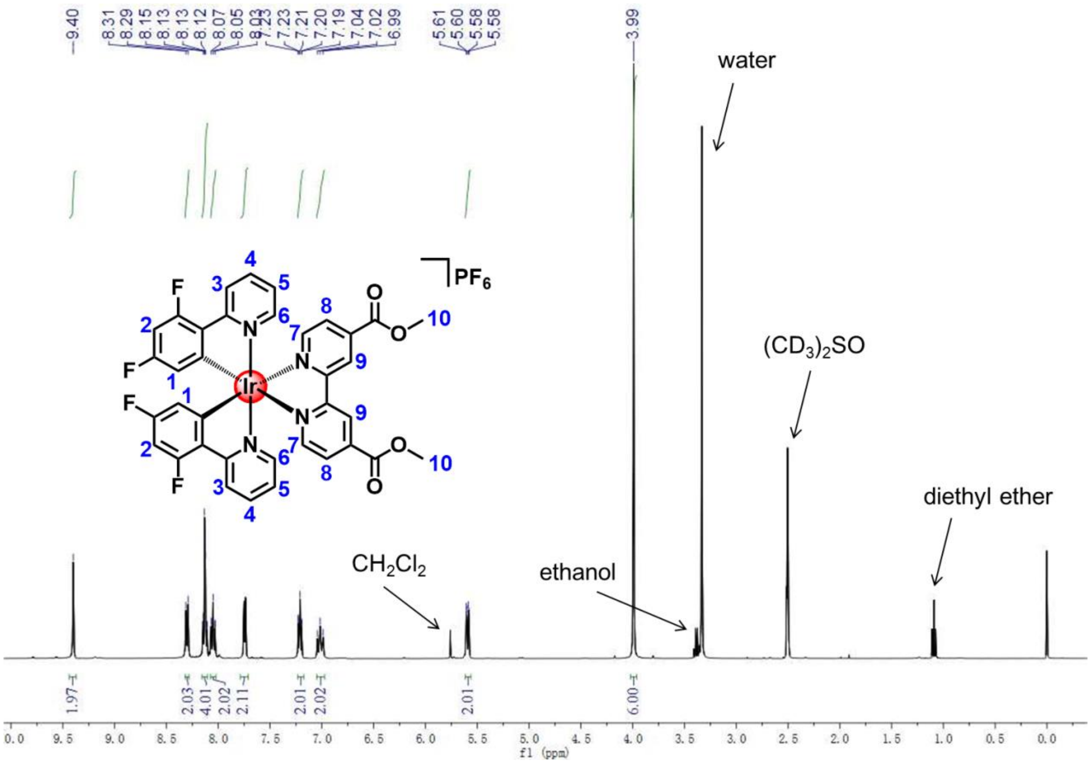

Figure S8. ^{1}H NMR spectrum of **2b**.

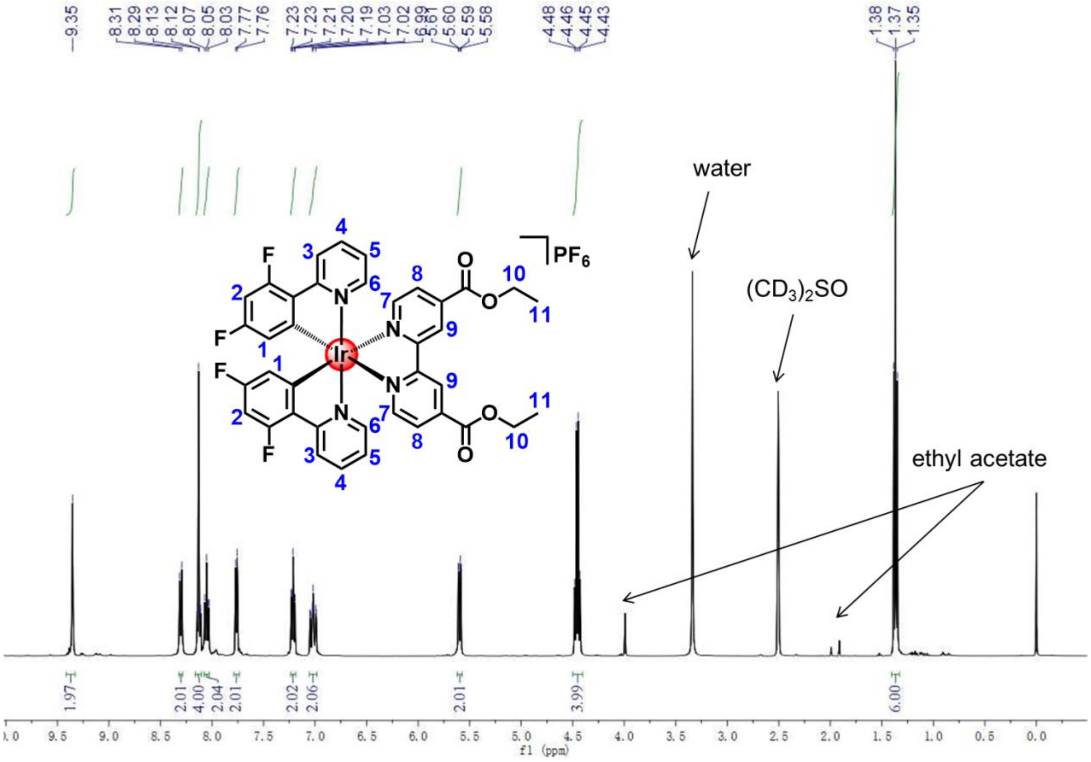

Figure S9. ^{1}H NMR spectrum of **3b**.

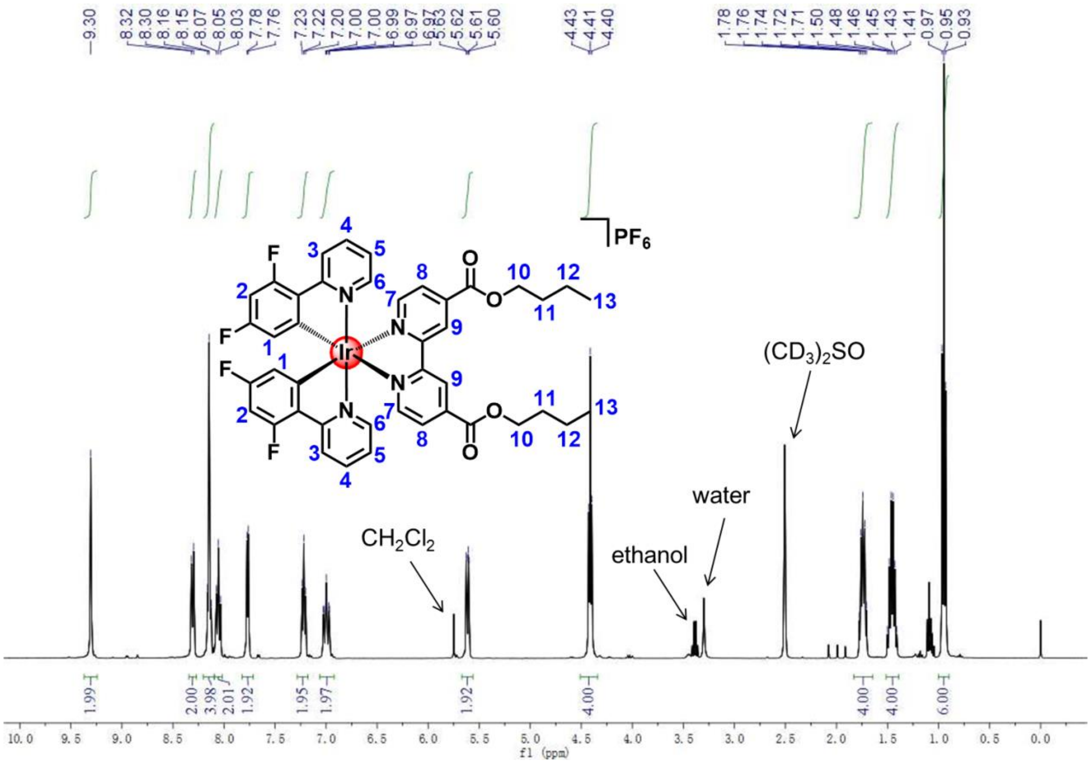

Figure S10. ^{1}H NMR spectrum of **4b**.

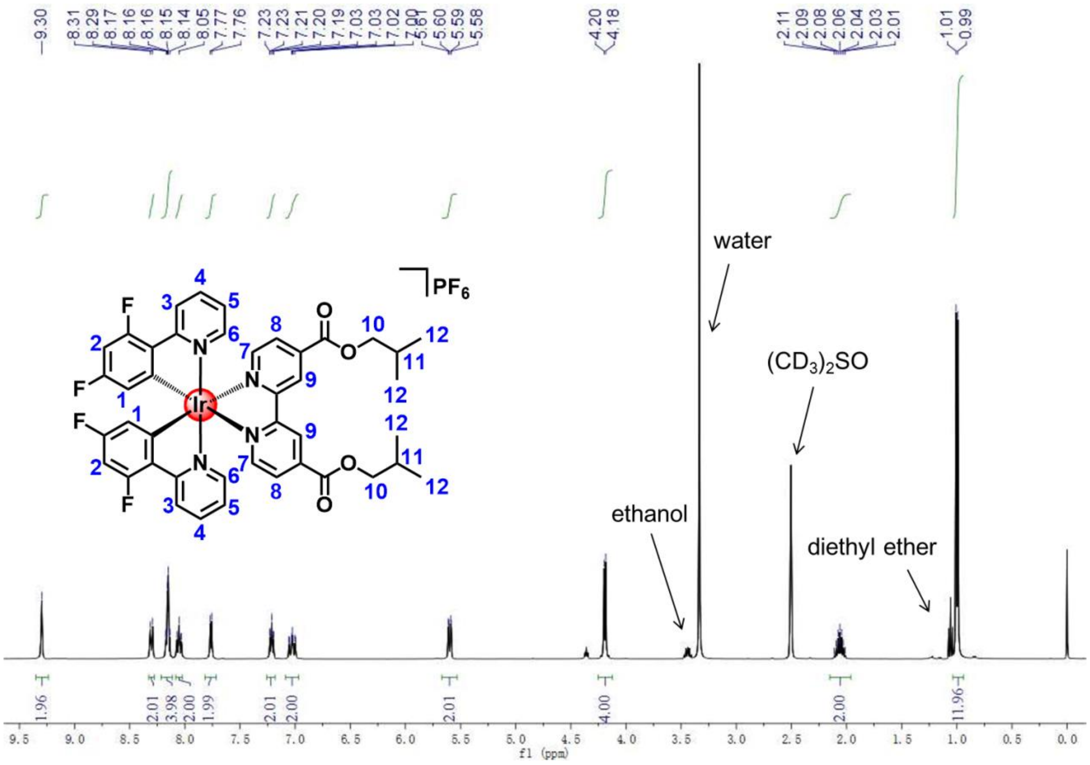

Figure S11. ^{1}H NMR spectrum of **5b**.

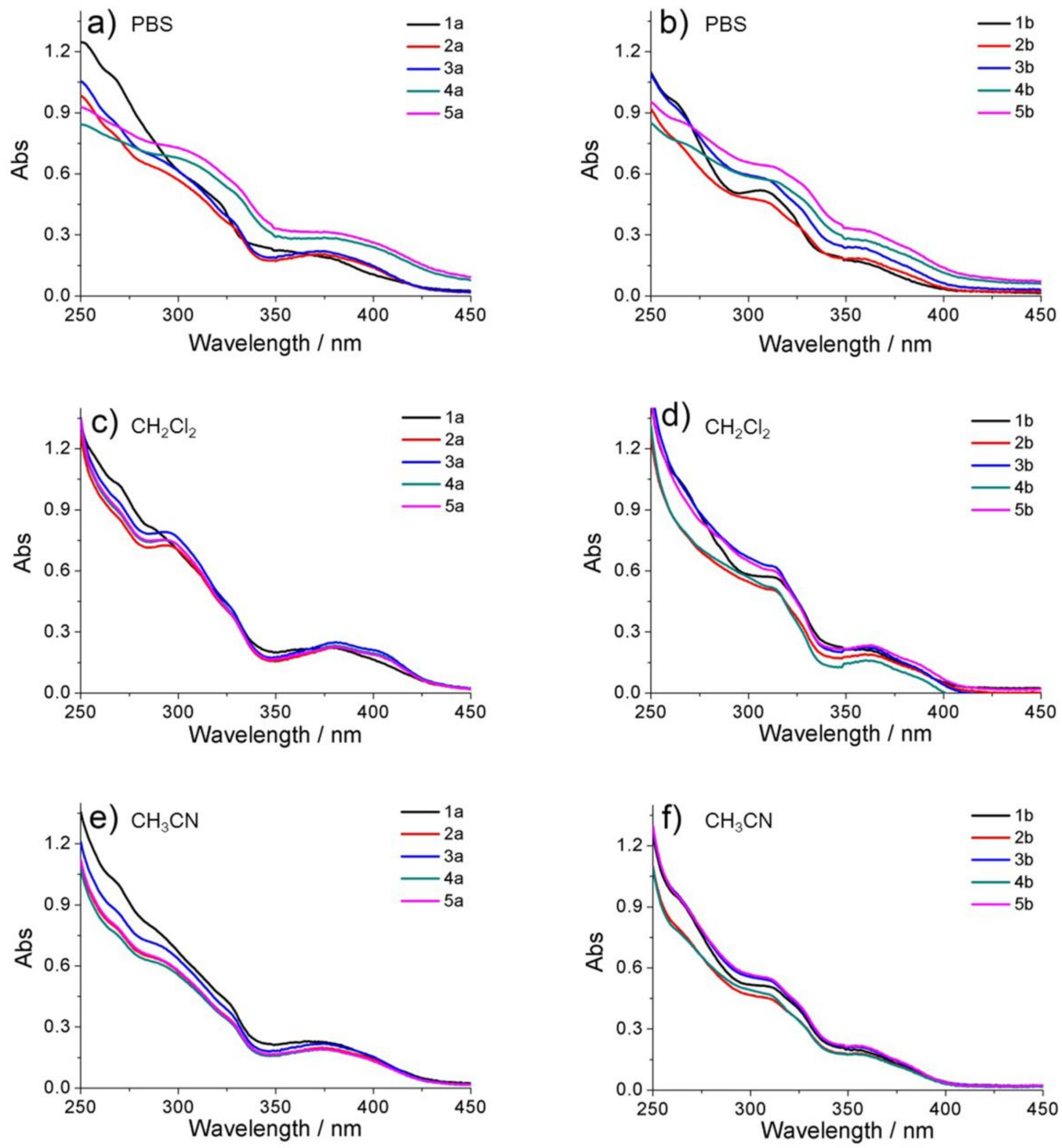

Figure S12. UV-vis spectra of Ir(III) complexes (20 μM) measured in PBS (a and b), CH_2Cl_2 (c and d) and CH_3CN (e and f) at 298 K.

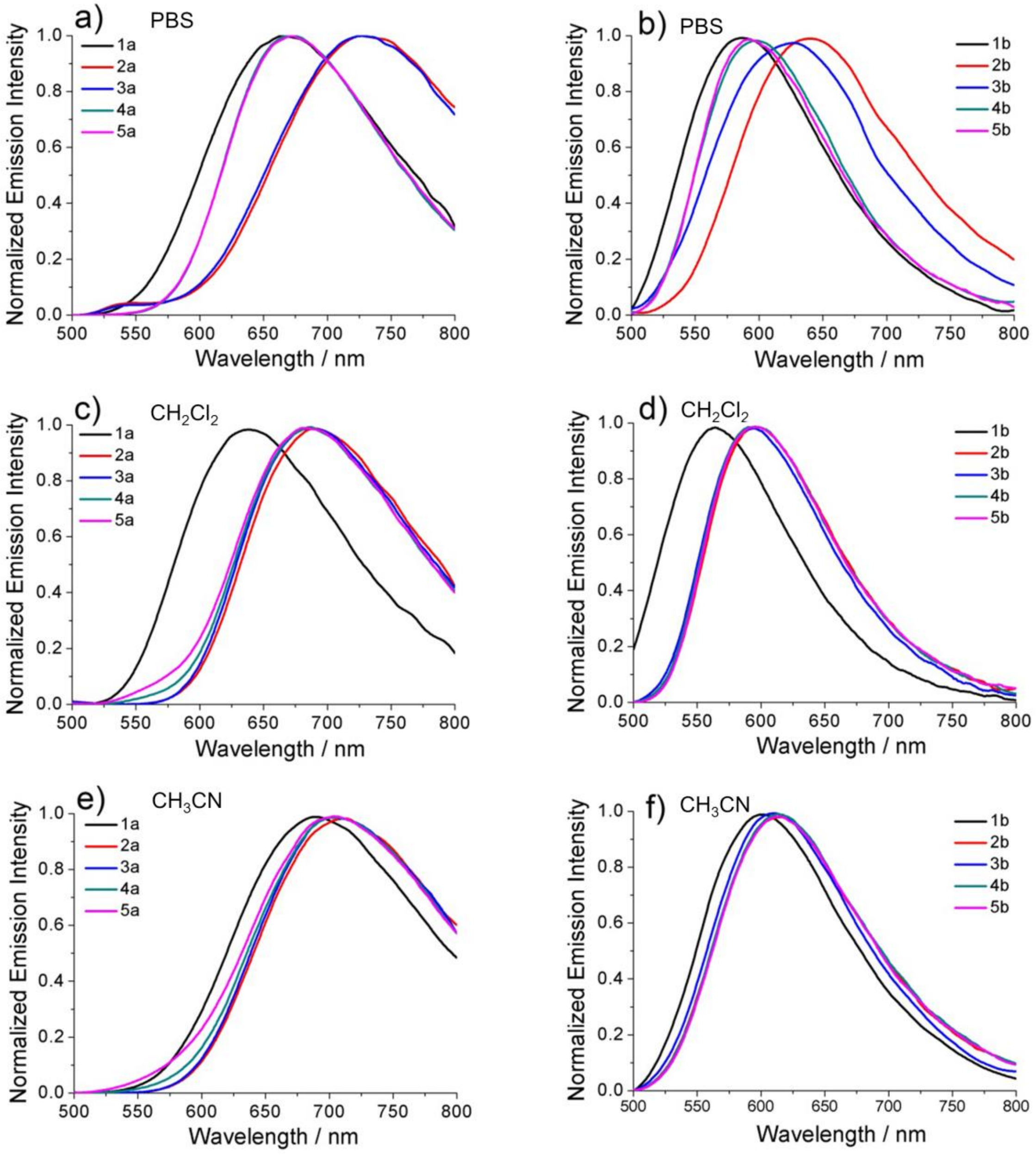

Figure S13. Emission spectra of Ir(III) complexes (20 μM) measured in PBS (a and b), CH_2Cl_2 (c and d) and CH_3CN (e and f) at 298 K (λ_{ex} = 405 nm).

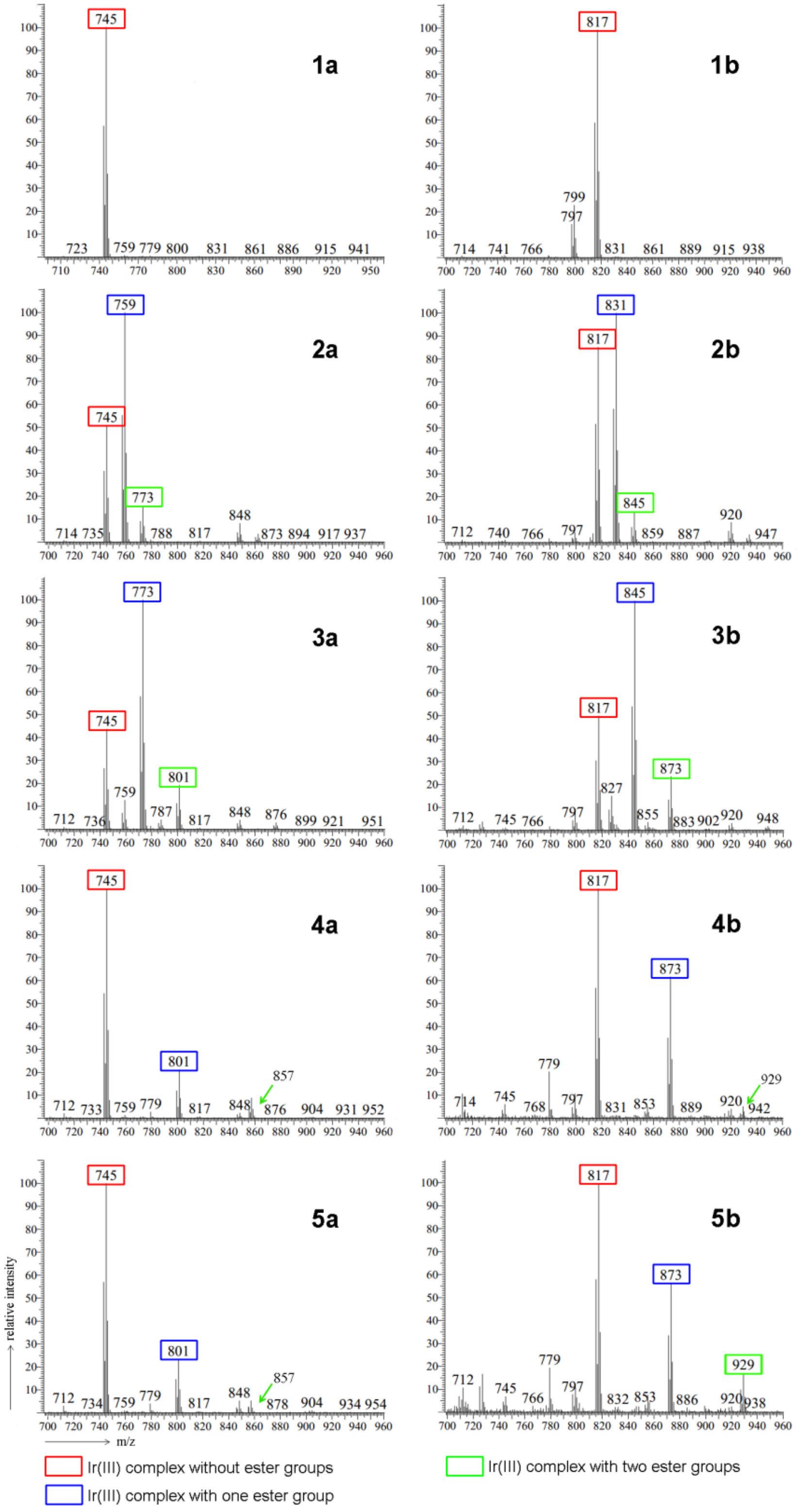

Figure S14. Mass spectra of Ir(III) complexes (20 μM, 100 μL) after hydrolysis by PLE in Tris-HCl buffer (10 mM, pH 7.4) at 298 K for 2 h.

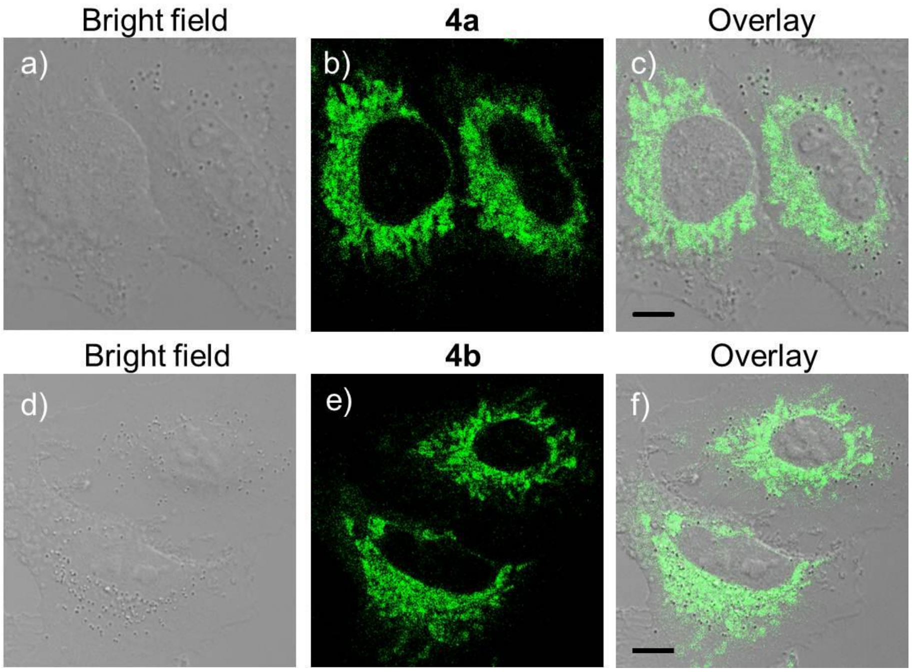

Figure S15. Confocal images of A549 cells after incubation with **4a** (5 μM) or **4b** (5 μM) for 30 min at 310 K. The excitation wavelength of Ir(III) complexes was 405 nm. Emission was collected at 630–690 nm (**4a**) and 540–600 nm (**4b**).

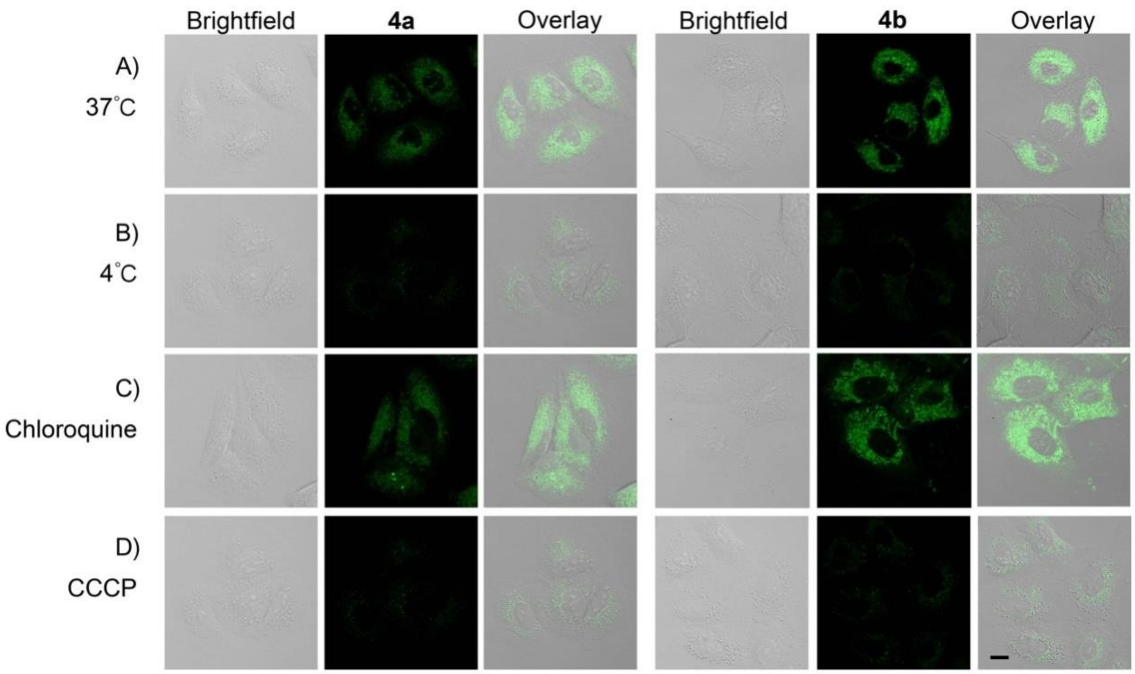

Figure S16. Confocal images of A549 cells after incubation with **4a** (5 μM) and **4b** (5 μM) under different conditions. A) Cells were incubated with Ir(III) complex at 310 K for 30 min. B) Cells were incubated with Ir(III) complex at 277 K for 30 min. C) Cells were pre-incubated with chloroquine (50 μM) for 1 h, and then incubated with Ir(III) complex for 30 min at 310 K. D) Cells were pre-incubated with CCCP (10 μM) for 1 h and then incubated with Ir(III) complex for 30 min at 310 K. The excitation wavelength of Ir(III) complexes was 405 nm. Emission was collected at 630–690 nm (**4a**) and 540–600 nm (**4b**).

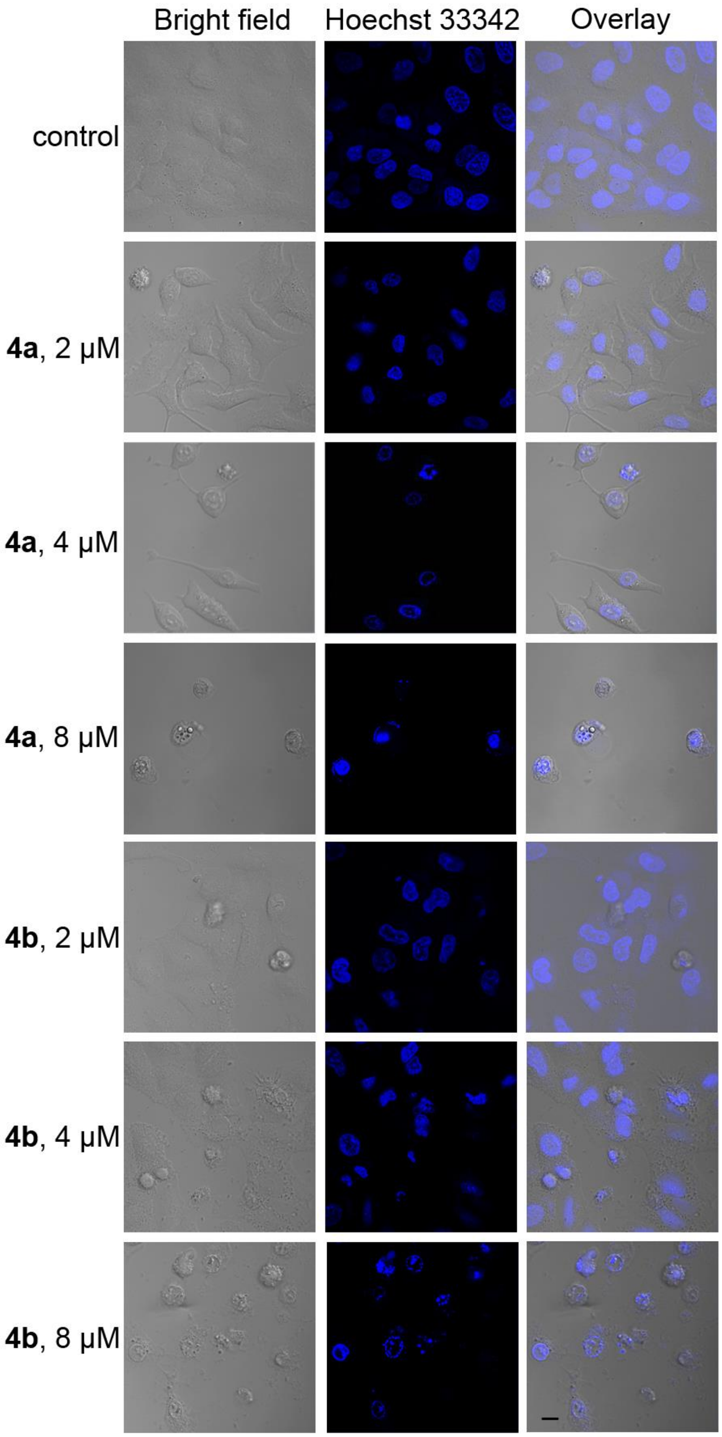

Figure S17. Confocal microscopic analysis of morphological alterations of A549 cells treated with **4a** or **4b** for 24 h. Cells were stained with Hoechst 33342. The excitation wavelength was 405 nm, while emission was collected at 440–480 nm.

Table S1. Crystallographic data of **2a** and **2b**

Complex	**2a**	**2b**
CCDC deposition no.	1469086	1469087
Empirical formula	$C_{36}H_{28}F_6N_4PIrO_4$	$C_{36}H_{24}F_{10}N_5PIrO_4$
Molecular weight	917.79	989.76
Description	Red	Yellow
Temperature (K)	298(2)	298(2)
λ (Å)	0.71073	1.54178
Crystal system	monoclinic	monoclinic
Space group	*P*21/*n*	*P*21/*n*
a (Å)	9.2306(2)	9.6802(2)
b (Å)	30.1850(9)	30.1669(6)
c (Å)	12.1649(3)	12.1428(3)
α (^{o})	90	90
β (^{o})	96.208(2)	95.288(2)
γ (^{o})	90	90
Volume, $Å^3$	3369.58(15)	3530.87(13)
Z	4	4
Absorption coefficient (mm^{-1})	4.091	8.657
F(000)	1800	1928
θ_{max} (deg)	29.385	73.726
Completeness to θ_{max}	99.9%	99.9%
Density(calcd)(mg/m^{-3})	1.809	1.862
[R_{int}]	0.0519	0.0733
Reflections collected/unique	21021/8054	12469/6903
$R1^a[I > 2\sigma(I)]$	0.0391	0.0624
$wR2^a$	0.0671	0.1563
GOF^b	1.128	1.085

$^aR1 = \sum\|F_0|-|F_c\|/\sum|F_0|, wR2 = \left\{\sum\left[w\left(F_0^2 - F_c^2\right)^2\right]/\sum\left[w\left(F_0^2\right)^2\right]\right\}^{1/2}$ $^bGOF = \left\{\sum\left[w\left(F_0^2 - F_c^2\right)^2/(n-p)\right]\right\}^{1/2}$

Where *n* is the number of data and *p* is the number of parameters refined.

Table S2. Selected bond lengths (Å) and bond angles (deg) of **2a** and **2b**

Complex	**2a**		**2b**	
Bond lengths (Å)	Ir1–N1	2.044(3)	Ir1–N1	2.057(6)
	Ir1–N2	2.047(3)	Ir1–N2	2.062(7)
	Ir1–N3	2.134(4)	Ir1–N3	2.130(7)
	Ir1–N4	2.134(3)	Ir1–N4	2.136(6)
	Ir1–C1	2.014(4)	Ir1–C1	2.015(8)
	Ir1–C12	2.013(4)	Ir1–C12	2.021(7)
Bond angles (deg)	C12–Ir1–C1	89.72(16)	C1–Ir1–C12	90.5(3)
	C1–Ir1–N1	80.77(14)	C1–Ir1–N1	80.3(3)
	C12–Ir1–N2	80.63(15)	C12–Ir1–N2	80.6(3)
	N3–Ir1–N4	76.41(13)	N3–Ir1–N4	76.4(2)

Table S3. Photophysical properties of iridium(III) complexes in different solutions[a]

Medium	Complex	$\lambda_{ex}/\lambda_{em}$	Φ_{em}[b]	τ[c]	Complex	$\lambda_{ex}/\lambda_{em}$	Φ_{em}[b]	τ[c]
PBS		352/660	0.005	14.0		350/568	0.082	162.8
CH_2Cl_2	**1a**	379/620	0.057	231.4	**1b**	363/563	0.381	636.0
$CNCH_3$		367/690	0.190	60.8		353/603	0.426	233.0
PBS		380/692	0.002	6.1		359/620	0.042	73.5
CH_2Cl_2	**2a**	365/710	0.033	91.4	**2b**	360/600	0.403	625.3
$CNCH_3$		374/740	0.038	36.3		354/610	0.412	277.1
PBS		374/740	0.001	4.4		360/615	0.017	105.8
CH_2Cl_2	**3a**	381/690	0.032	100.0	**3b**	360/595	0.356	687.7
$CNCH_3$		368/710	0.030	39.7		353/605	0.394	285.7
PBS		375/660	0.001	28.1		360/600	0.021	310.6
CH_2Cl_2	**4a**	381/690	0.038	100.7	**4b**	360/595	0.440	689.6
$CNCH_3$		368/700	0.041	49.8		354/610	0.457	291.0
PBS		375/670	0.001	51.7		360/590	0.031	341.5
CH_2Cl_2	**5a**	381/680	0.036	106.2	**5b**	360/598	0.464	700.8
$CNCH_3$		368/700	0.045	49.5		354/610	0.452	285.3

[a]All emission decays were obtained on freshly prepared samples (20 μM) in quartz cuvettes at room temperature. [b] Solutions of $[Ru(bpy)_3](PF_6)_2$ were used as standard, PBS (Φ_{em} = 0.042)[1], CH_2Cl_2 (Φ_{em} = 0.059)[2] and CH_3CN (Φ_{em} = 0.062)[3]. [c] Decay curves of complexes were recorded by an Edinburgh FLS 920 Spectrometer. The lifetimes were measured at the maximal emission wavelength.

REFERENCES

(1) Houten, J. V. & Watts, R. J. Temperature Dependence of the Photophysical and Photochemical Properties of the Tris(2,2'-bipyridyl)ruthenium(II) Ion in Aqueous Solution. *J. Am. Chem. Soc.* **98**, 4853-4858 (1976)

(2) Pucci, D. *et al.* Room temperature columnar mesomorphism and high quantum yield phosphorescence in ionic ruthenium(II) 2,2'-bipyridine-based complexes. *J. Mater. Chem.* **19**, 7643-7649 (2009).

(3) Tyson, D. S. & Castellano, F. N. Intramolecular Singlet and Triplet Energy Transfer in a Ruthenium(II) Diimine Complex Containing Multiple Pyrenyl Chromophores. *J. Phys. Chem. A* **103**, 10955-10960 (1999).
